# OTUD4-mediated GSDME deubiquitination enhances radiosensitivity in nasopharyngeal carcinoma by inducing pyroptosis

**DOI:** 10.1186/s13046-022-02533-9

**Published:** 2022-11-21

**Authors:** Muping Di, Jingjing Miao, Qiuzhong Pan, Zonglong Wu, Boyu Chen, Muru Wang, Jingjing Zhao, Huageng Huang, Jiewen Bai, Qijing Wang, Yan Tang, Yongqiang Li, Jia He, Tong Xiang, Desheng Weng, Lin Wang, Jianchuan Xia, Chong Zhao

**Affiliations:** 1 Sun Yat-Sen University Cancer Center, State Key Laboratory of Oncology in South China, Collaborative Innovation Center for Cancer Medicine, Guangzhou, 510060 China; 2 Department of Nasopharyngeal Carcinoma, Guangdong Key Laboratory of Nasopharyngeal Carcinoma Diagnosis and Therapy, Sun Yat-Sen University Cancer Center, Guangzhou, 510060 China; 3 Department of Biotherapy, Sun Yat-Sen University Cancer Center, Guangzhou, 510060 China; 4 Department of Urology, Peking University Third Hospital, Beijing, 100000 China; 5 Division of Gastroenterology, Tongji Hospital, Tongji Medical College, Huazhong University of Science and Technology, Wuhan, 430030 China

**Keywords:** OTUD4, GSDME, Pyroptosis, Radioresistance, Nasopharyngeal carcinoma

## Abstract

**Background:**

Radioresistance is the primary cause of nasopharyngeal carcinoma (NPC) treatment failure. Previous studies have focused on the deficits in cellular apoptosis as a mechanism for radioresistance; however, additional potential death modes involved in modulating radiosensitivity of NPC have not been explored.

**Methods:**

Pyroptosis was assessed by phase-contrast imaging, LDH release assays, live cell imaging, and Western blotting. In vitro and in vivo assays were used to investigate the function of gasdermin E (GSDME) and ovarian tumor family deubiquitinase 4 (OTUD4). NPC tissues were analyzed using Western blotting, immunohistochemistry, and real-time PCR. The molecular mechanism was determined using immunoprecipitation assays and mass spectrometry.

**Results:**

Live cell imaging revealed that 40—75% of irradiation-induced dead NPC cells were pyroptotic cells. Furthermore, irradiation-induced pyroptosis is triggered by GSDME, which are cleaved by activated caspase-3 in the intrinsic mitochondrial pathway. Additionally, GSDME was significantly downregulated in radioresistant NPC specimens. Low GSDME expression was a predictor of worse prognosis and conferred NPC radioresistance both in vitro and in vivo. Mechanistically, OTUD4 deubiquitinated and stabilized GSDME, enhancing radiosensitivity of NPC cells by promoting pyroptosis. Clinically, OTUD4 was significantly correlated with GSDME in NPC biopsies, and patients with low expression of both OTUD4 and GSDME suffered the worst radiotherapy response and survival.

**Conclusions:**

GSDME-dependent pyroptosis is a critical determinant of radiosensitivity in NPC, and is modulated by OTUD4 via deubiquitinating and stabilizing GSDME. These findings reveal a promising novel direction to investigate radioresistance and suggest potential therapeutic targets for sensitizing NPC to radiotherapy.

**Supplementary Information:**

The online version contains supplementary material available at 10.1186/s13046-022-02533-9.

## Background

Radiotherapy (RT) is the most common treatment for nasopharyngeal carcinoma (NPC), a malignant tumor which derives from the nasopharyngeal epithelium [1]. Due to the widespread application of intensity-modulated radiation therapy (IMRT), the locoregional disease control and overall survival of patients with NPC have considerably improved [2]. Nevertheless, 10–17% of NPC patients suffer local recurrence, even in early-stage NPC [3, 4]. Radioresistance is a major barrier to the successful treatment of NPC [5]. Therefore, uncovering the underlying mechanisms of radioresistance in NPC is crucial.

Pyroptosis, an inflammatory programmed cell death, is executed by the gasdermin protein family [6–10]. Gasdermin family members contain a similar amino (N)-terminal domain structure, possessing pore-forming activity when cleaved by cleaved-caspases or granzymes [11, 12]. Apoptosis has been widely recognized and studied as a mechanism for regulating cell death in antitumor treatment responses over the past few decades [13, 14]. Recently, growing evidence has confirmed the antineoplastic effects of pyroptosis in chemotherapy [9, 15–19], targeted therapy [20], and immunotherapy [21]. Pyroptosis differs from apoptosis in that it involves cytolysis, as indicated by large bubbles erupting from a swollen cell membrane, which liberates cell contents, including various inflammatory molecules, resulting in the activation of antitumor immunity [10, 22]. Moreover, pyroptosis triggered by chemotherapy drugs and radiotherapy was found to be associated with the toxicity of chemotherapy drugs and radiation injury in normal tissues [9, 23–25]. Pyroptosis and apoptosis are two different ways of cell death [26]. The proportion of dead cells after irradiation represents the radiosensitivity of cancer cells. Thus, pyroptosis probably affects the radiosensitivity of NPC cells. Nevertheless, the role of pyroptosis in NPC radiotherapy remains elusive.

Ubiquitination and deubiquitination are vital posttranslational modifications regulating various critical cellular processes [27]. Deubiquitination is the functionally reciprocal process of ubiquitination that prevents protein proteasomal degradation through deubiquitinating enzymes (DUB)-mediated removal of ubiquitin chains from target proteins [28]. Ovarian tumor family deubiquitinase 4 (OTUD4), a member of the OTU family, preferentially cleaves K48-conjugated polyubiquitin chains and participates in the deubiquitination of multiple proteins [29–31]. Viral infection causes the IRF3/7-dependent induction of OTUD4, which binds to MAVS to cleave K48-conjugated ubiquitin chains, stabilizing MAVS and activating antiviral innate immune responses [31]. Recently, OTUD4 has been identified to be down-regulated in multiple human tumor types, and lower OTUD4 expression indicates a poor prognosis [32]. However, the expression and functional role of OTUD4 in NPC remain largely unknown.

In this study, we identified that radiotherapy induced gasdermin E (GSDME)-dependent pyroptosis in NPC cells through the intrinsic mitochondrial apoptotic pathway. Low GSDME expression could predict worse prognosis and was associated with radioresistance both in vitro and in vivo. In addition, we demonstrated that OTUD4 deubiquitinated and stabilized GSDME, enhancing radiosensitivity in NPC by promoting GSDME-dependent pyroptosis. These novel findings indicate a possible direction to investigate radiosensitivity, and open new avenues to sensitize NPC to radiotherapy by targeting pyroptosis.

## Materials and methods

### Cell culture

Sun Yat-sen University Cancer Center (State Key Laboratory of Oncology in South China, Guangzhou, China) provided the NPC cell lines HK1, SUNE1, HONE1, HNE1, 5-8F, 6-10B and CNE1, melanoma cell lines A375 and SK-MEL-30, esophageal cancer cell lines KYSE30 and KYSE250, prostate cancer cell lines PC3 and 22RV1, as well as breast cancer cell lines 159 and 231. Cells were maintained in Roswell Park Memorial Institute (RPMI) 1640 medium (Invitrogen, Carlsbad, CA, USA) containing 10% fetal bovine serum (FBS) (HyClone, Logan, UT, USA) plus 1% penicillin/streptomycin (Invitrogen, Carlsbad, CA, USA). HEK293T cells were maintained in Dulbecco’s modified Eagle’s medium (DMEM) (Gibco, Grand Island, NY, USA) supplemented with 10% FBS. All cells were cultured and maintained in a humidified atmosphere with 5% CO2 incubator at 37 °C.

### Clinical tissue samples and patient information

The Institutional Review Board of Sun Yat-Sen University Cancer Center approved the present study (Approval No. GZR2020-193). Between 2011 and 2015, 150 paraffin-embedded tumor specimens were obtained from pathologically diagnosed, non-metastatic NPC patients who were treated with radical radiotherapy or combined chemoradiotherapy at the Sun Yat-Sen University Cancer Center. Serum LDH levels were measured by the clinical laboratory of Sun Yat-sen University Cancer Center. Nasopharyngoscopy images and magnetic resonance images were provided by the Nasopharyngeal Carcinoma Department and Medical Imaging Department, respectively. Radiosensitive NPC patients were outlined as ones with complete regression after radical radiotherapy or without relapse after the end of radiation therapy. Radioresistant NPC patients were outlined as ones with incomplete regression of lesions after radical radiotherapy; residual tumors at more than six weeks after the end of radiation therapy; or relapse after radical radiation therapy. The fresh NPC samples were quickly frozen in liquid nitrogen and stored at -80 °C until use. The detailed clinical information of the samples is summarized in Supplementary Table S1.

### Immunohistochemistry (IHC)

Immunohistochemical staining was performed using GSDME antibody (#ab175614, Abcam) and OTUD4 antibody (#HPA036623, Sigma) on 150 paraffin-embedded NPC tissue sections. The stained tissues were examined by two independent pathologists. Evaluation Principles included the proportion of tumor cells and staining intensity. The proportion of tumor cells was scored as follows: 0 (no positive tumor cells); 1 (< 10% positive tumor cells); 2 (10–35% positive tumor cells); 3 (35–75% positive tumor cells); 4 (> 75% positive tumor cells). The staining intensity was graded according to the following criteria: 1 (absent or weak staining = light yellow); 2 (moderate staining = yellow brown); 3 (strong staining = brown). The staining index (SI; values 0‐12) = the proportion of tumor cells × staining intensity. Tissues with an SI ≥ 6 were divided into high expression, while tissues with an SI score below 6 were divided into low expression. We used the Kaplan–Meier method to estimate locoregional recurrence-free survival (LRRFS) and progression-free survival (PFS); the log-rank test to assess survival differences between groups.

### Plasmids, transfection, and siRNA knockdown

NPC cells were infected using lentivirus generated by the pEGFP-N1 vector for 72 h, and the GFP-positive cells were collected on MoFlo Astrios (Beckman Coulte). Polymerase chain reaction (PCR) was used to amplify the cDNA fragments of GSDME and OTUD4, which was then cloned into vector pLVX-IRES-Hyg. Short-hairpin RNAs (shRNAs) targeting GSDME and OTUD4 were cloned into the vector pSuper-retro-neo. Stable cell lines with GSDME/OTUD4 overexpression and GSDME/OTUD4 knockdown were generated via infection of retrovirus packaged from HEK293T cells. The viral supernatants were infected into target cells for 72 h, and then corresponding antibiotics were used to select stable cell lines for 9 days.

Specific siRNAs (5′-GATGATGGAGTATCTGATCTT-3′ and 5′-GGAUUGAUGAGGAGGAAUUTT-3′) were used to knock down GSDME and GSDMD expression respectively, following the manufacturer's protocol. The efficiency of depletion was confirmed by Western blotting. shRNA target sequences are listed in Supplementary Table S4.

### Microscopy

To observe the morphology of pyroptotic, apoptotic, and surviving cells, cells were seeded in dishes at an appropriate density and received the indicated treatments. Cell images were acquired using an Olympus IX73 microscope. To capture the dynamic process of cell death, EGFP-expressing cells were seeded up to appropriate density into a 35 mm glass-bottom culture dishes, and then videos were taken using a CV1000 Confocal Scanner System (Yokogawa, Tokyo, Japan) in 50 randomly selected fields of view. Pyroptotic, apoptotic, and surviving cells were counted and analyzed using CV1000 Software.

### Flow cytometry

Cells were subjected to the indicated treatments, and then were collected, and stained with the Annexin V-Alexa647 and propidium iodide (PI) Apoptosis Kit (Yishan Biotech) following the manufacturer’s instructions. Stained cells were analyzed on a FACSort instrument (BD Biosciences) with CellQuest software.

### Cell viability assay

The 3-(4,5-Dimethyl-2-thiazolyl)-2,5-diphenyl-2H-tetrazolium bromide (MTT) kit (Sigma) was used to measure cell viability following the manufacturer’s instructions.

### Western blotting

Cells and tissue samples were lysed in RIPA buffer with proteinase inhibitors (Sigma) and phosphatase inhibitors (Sigma). Cellular fractionation was performed using the Qproteome Cell Compartment Kit (Qiagen). Proteins were detected using the following primary antibodies: GSDME (#ab215191, Abcam), GSDMD (#ab210070, Abcam), Caspase-3 (#9665, CST), PARP (#9532, CST), cytochrome c (#11,940 S, CST), OTUD4 (#SAB3500018, Sigma), Myc tag (#2278S, CST), Flag tag (#14793S, CST), HA tag (#3724, CST), and GAPDH (#5174, CST). The secondary antibodies used were horseradish peroxidase-conjugated anti-mouse or anti-rabbit antibodies (GE Healthcare). The secondary antibody anti-rabbit IgG light chain specific (#211–032-171, Jackson Immuno Research Laboratories) was used for immunoprecipitation assays to weaken the signals from the heavy chain.

### Immunoprecipitation (IP) assays

Whole-cell lysates were prepared using IP lysis buffer (Beyotime, Wuhan, China) and precipitated with anti-Flag-tag beads (Sigma-Aldrich) or anti-Myc-tag beads (Biolegend) overnight at 4 °C. Precipitates were washed 6 times with cold IP wash buffer. Elutes were further subjected to Western blotting or mass spectrometry (MS) analysis.

### LDH release assay

Following the indicated treatments, the release of LDH was determined using an LDH assay kit (Solarbio) according to the manufacturer’s protocol.

### Mouse models

Male BALB/c nude mice (3–4 weeks) were purchased from the Experimental Animal Center of Sun Yat-sen University (Guangzhou, China). Mice were divided into four groups randomly. 1 × 10^6^ indicated cells were then subcutaneously injected into the nude mice. When tumor volumes reached 50–100 mm^3^, the mice in the radiotherapy group were irradiated with 12 Gy (2 Gy/day for 6 days). Tumor size was monitored every 4 days, and the tumor volume was calculated as: volume = 1/2 × L × W^2^. Blood samples were collected, and serum LDH concentrations were evaluated pre-radiotherapy and following the third and sixth radiotherapy treatments. The tumor burdens of nude mice were measured using IVIS Lumina II with (Caliper) Living Image Software on day 42. Tumors were then excised from euthanized mice, weighed, and paraffin-embedded. Above paraffin-embedded samples were sectioned for IHC and hematoxylin–eosin (H&E) staining. All the animal experiments in this study were authorized by the Institutional Animal Care and Use Committee of the Sun Yat-sen University Cancer Center (SYSUCC) (Approval No. L102012021110I).

### Statistical analysis

SPSS version 19.0 (IBM Corp., Armonk, NY, USA) and GraphPad Prism software were applied in the statistical analysis. The statistical tests for data analysis included the χ^2^ test, two-tailed Student’s t test, log-rank test, Spearman-rank correlation test, and one-way or two-way ANOVA. Data are expressed as the mean ± SD. Significant difference was defined by *p ≤ 0.05, **p ≤ 0.01, ***p ≤ 0.001, and ****p ≤ 0.0001.

## Results

### Radiotherapy induces GSDME-dependent pyroptosis in NPC cells through the intrinsic mitochondrial apoptotic pathway

**Fig. 1:**
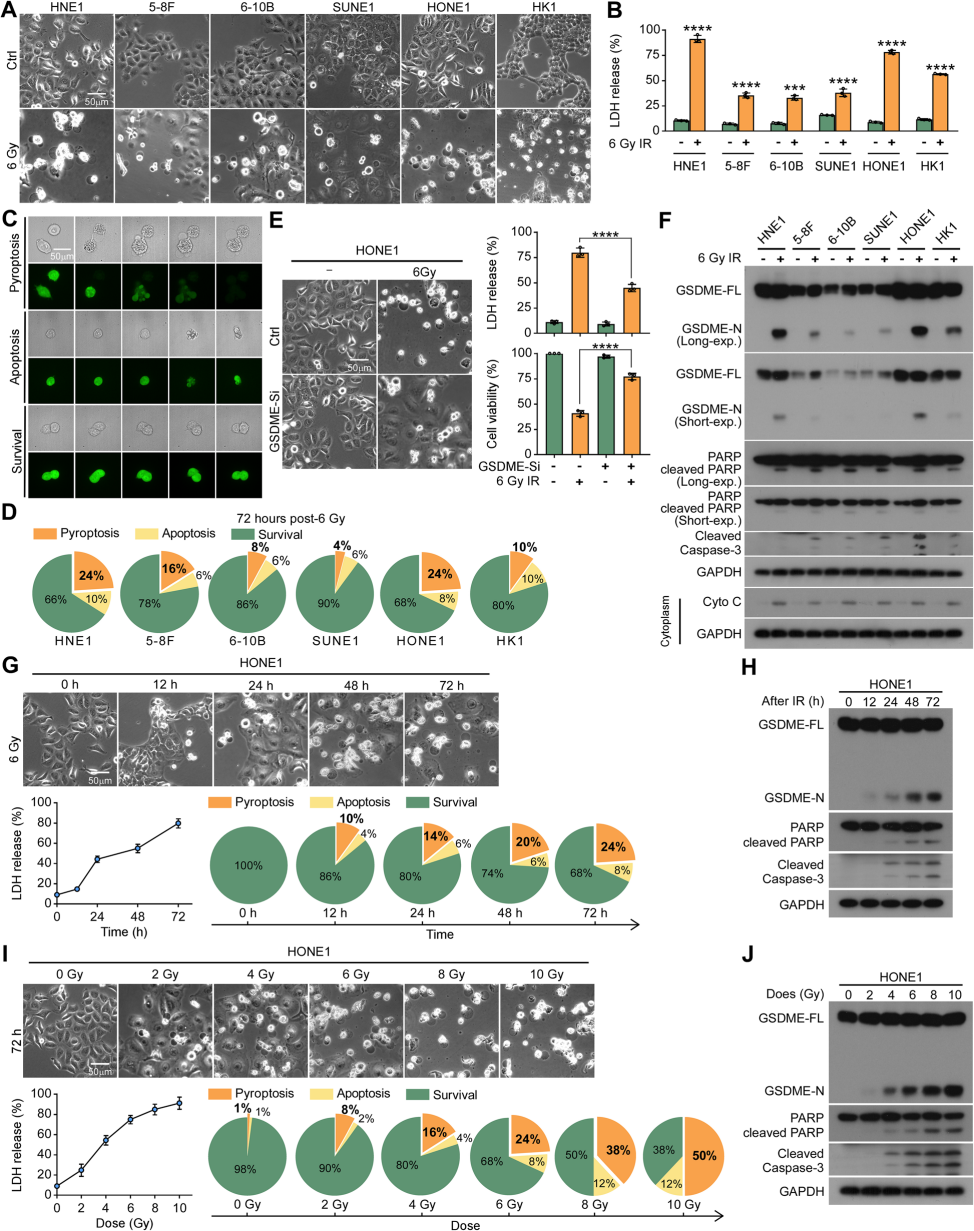
Radiotherapy induces GSDME-dependent pyroptosis in NPC cells through the intrinsic mitochondrial apoptotic pathway. **A-B** Six NPC cell lines were treated with or without 6 Gy irradiation. 72 h later, (**A**) cell morphological changes and (**B**) LDH release were measured. **C** Typical processes of cell pyroptosis, apoptosis and survival in HNE1 cells are shown. Brightfield and fluorescent images of stable EGFP-overexpressing NPC cells were taken at specific time-points post-6 Gy irradiation. **D** Percentage of pyroptotic, apoptotic, and viable cells in six NPC cells lines within 72 h after 6 Gy irradiation is indicated. **E** GSDME silencing inhibited pyroptosis (left), LDH release (upper right), and cell death (bottom right) in HONE1 cells at 72 h after 6 Gy irradiation. **F** Western blotting revealed the levels of indicated proteins in six NPC cell lines at 72 h after 6 Gy irradiation. Cyto C: cytochrome c. **G** Cell morphological changes (top), LDH release assay (bottom left), live cell imaging (bottom right), and (H) the levels of indicated proteins were assessed in HONE1 cell lines at the indicated time points after irradiation (6 Gy). (I) Cell morphological changes (top), LDH release assay (bottom left), live cell imaging (bottom right), and (J) the levels of indicated proteins were assessed in HONE1 cell lines at 72 h after the indicated irradiation dose. GAPDH was used to normalize the amount of protein loaded. All data are presented as the mean ± SD of three independent experiments. ns, No significant difference. ***P < 0.001, ****P < 0.0001

It is generally accepted that radiation destroys cancer cells primarily by inducing apoptosis [13]. In the present study, we observed that radiation caused the morphological characteristics of pyroptosis in NPC cells, as evidenced by swollen cells and the emergence of enormous bubbles from the plasma (Fig. 1A). The nature of cell death was further identified by increased LDH release (Fig. 1B). The necroptosis inhibitor GSK’872 could not reduce cell death, suggesting that pyroptosis instead of necroptosis was triggered by irradiation (Supplementary Figure S1A). Furthermore, the NPC cells stably expressing EGFP were irradiated at a dose of 6 Gy, and imaged using a CV1000 Confocal Scanner System. Supplementary Video 1–3 and Fig. 1C show typical processes of cellular pyroptosis, apoptosis and survival. In six NPC cell lines, the proportions of pyroptotic cells after irradiation ranged from 4 to 24% (Fig. 1D).

To identify which gasdermin family member is responsible for mediating radiation-induced pyroptosis in NPC cells, we knocked down the expression of GSDME and GSDMD by siRNAs in HONE1 cells (Supplementary Figure S1B and C). Knockdown of GSDME (Fig. 1E), but not GSDMD (Supplementary Figure S1D), attenuated typical morphological changes of pyroptosis, as well as the release of LDH and reduction in cell viability. Consistently, cleavage of GSDME was observed in NPC cells after radiation, accompanied by cleavage of caspase-3 and poly-(ADP-ribose) polymerase (PARP; a marker for cell apoptosis), and release of cytochrome c from mitochondria (Fig. 1F). In addition, after radiation exposure, the proportion of pyroptotic cells, the release of LDH, and the cleavage of GSDME, PARP, and caspase-3 increased in a time- and dose-dependent manner (Fig. 1G-J; Supplementary Figure S1E-H). The above data suggest that radiotherapy results in GSDME-dependent pyroptosis through the intrinsic mitochondrial apoptotic pathway.

### Upregulation of GSDME enhances pyroptosis and radiosensitivity in NPC cells in vitro

**Fig. 2:**
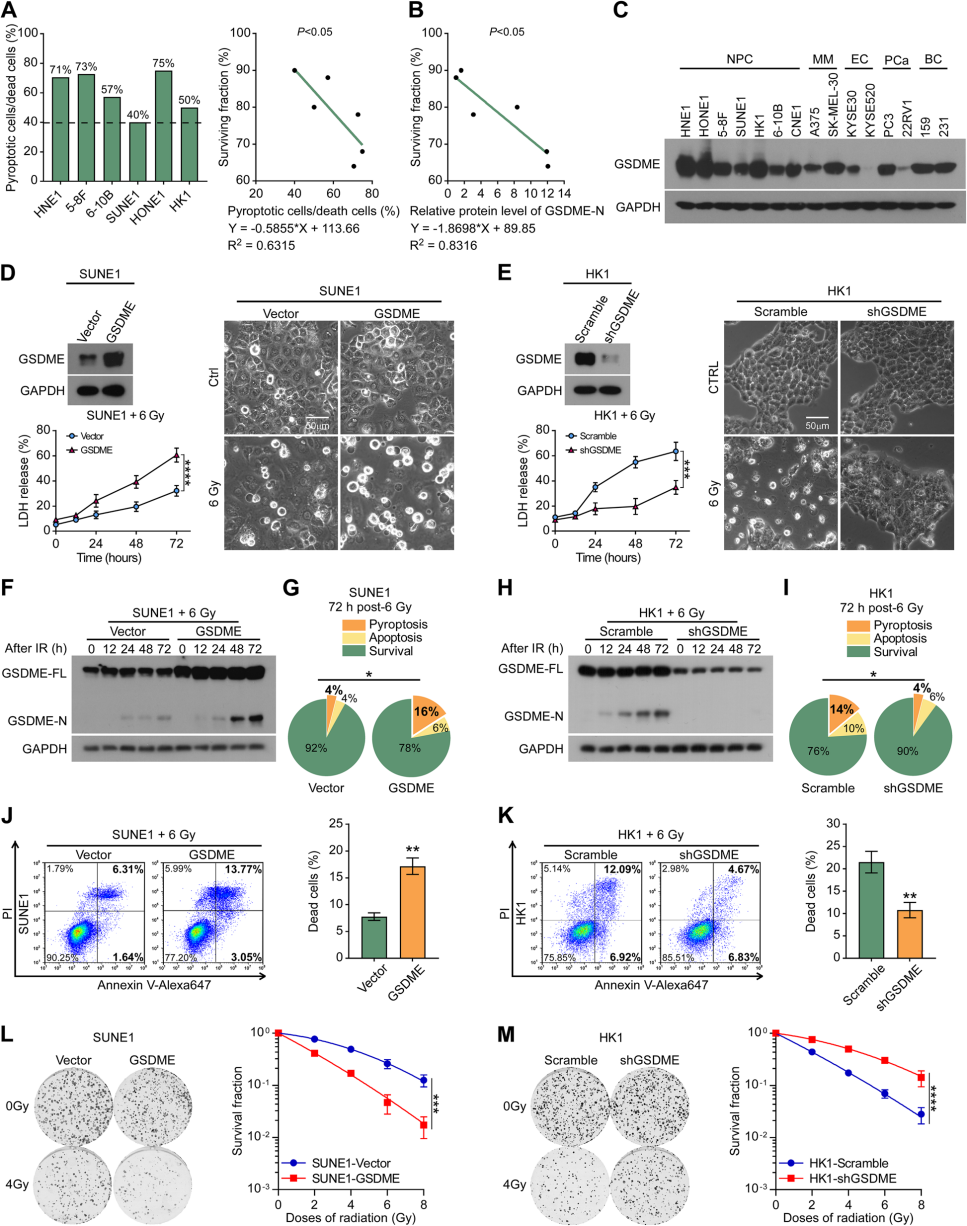
Upregulation of GSDME enhances pyroptosis and radiosensitivity in NPC cells in vitro. **A** The ratio of pyroptotic cells to dead cells in six NPC cell lines (HNE1, 5-8F, 6-10B, SUNE-1, HONE1, and HK1) within 72 h after 6 Gy irradiation, as calculated using live cell imaging data (left). Correlation analysis between the ratio of pyroptotic cells to dead cells and surviving fraction was performed (right). **B** Correlation analysis between relative GSDME-N levels and the surviving fraction in NPC cell lines within 72 h after 6 Gy irradiation was performed. **C** Western blotting showed GSDME expression in seven NPC cells and eight other cancer cells. MM: melanoma, EC: esophageal cancer, PCa: prostate cancer, BC: breast cancer. A375 cells were included as a positive control. **D-M** Stable GSDME-overexpressing SUNE1 cells (**D**, left upper) and stable GSDME-knockdown HK1 cells (**E**, left upper) were established. These cells were exposed to the indicated irradiation dose, and then phase-contrast cell imaging (**D**, E, right), LDH release assay (**D**, E, left lower), Western blotting analysis of GSDME-N (**F**, **H**), live cell imaging (**G**, **I**), Annexin/PI assay (**J**, **K**), and colony formation assay (**L**, **M**, left) were performed at designated time points. The survival curves of stable cell lines are indicated (**L**, **M**, right). GAPDH was used to determine the amount of loading proteins. All data are presented as the mean ± SD of three independent experiments. *P < 0.05, **P < 0.01, ***P < 0.001, ****P < 0.0001

Live cell imaging analysis showed that pyroptotic cells accounted for a large proportion of dead cells (≥ 40%) induced by radiation (Fig. 2A, left), and the proportion of pyroptotic cells/dead cells was inversely proportional to the surviving fraction (Y = -0.5855*X + 113.66, R^2^ = 0.6315) (Fig. 2A, right). Additionally, the relative protein level of GSDME-N was negatively correlated with the surviving fraction of NPC cell lines following 6 Gy irradiation (Y = -1.8698*X + 89.85, R^2^ = 0.8316) (Fig. 2B). GSDME is silenced in most tumors due to promoter methylation[33–35]. However, in this study, GSDME was found to be more highly expressed in NPC cell lines compared to the A375 melanoma cell line, which is known to express GSDME [36]. As representatives of radiosensitive tumors, NPC cells universally exhibited higher GSDME expression than other cancer cells (Fig. 2C).

To further assess the effect of GSDME on the radiosensitivity of NPC cells, we constructed SUNE1 and 6-10B cells that stably overexpress GSDME (Fig. 2D, left upper; Supplementary Figure S2A, left upper). Concurrently, we silenced endogenous GSDME expression in HK1 and HONE1 cells using shRNA (Fig. 2E, left upper; Supplementary Figure S2B, left upper). Upregulation of GSDME in SUNE1 and 6-10B cells led to more obvious pyroptotic bubbles (Fig. 2D, right; Supplementary Figure S2A, right), increased the release of LDH (Fig. 2D, left lower; Supplementary Figure S2A, left lower), enhanced the generation of GSDME-N fragments (Fig. 2F; Supplementary Figure S2C), resulted in a higher proportion of pyroptotic cells (Fig. 2G; Supplementary Figure S2D), and resulted in a higher proportion of dead cells after irradiation (Fig. 2J; Supplementary Figure S2K). On the other hand, downregulation of GSDME in HK1 and HONE1 cells resulted in the opposite effects (Fig. 2E, H, I, K; Supplementary Figure S2B, E, F, H). Furthermore, overexpression of GSDME enhanced the radiosensitivity of NPC cells (Fig. 2L; Supplementary Figure S2I), while knockdown of GSDME diminished radiosensitivity (Fig. 2M; Supplementary Figure S2J). Additionally, overexpression of GSDME increased the oligomerization level of GSDME-N in NPC cells after irradiation, while GSDME knockdown decreased the oligomerization level (Supplementary Figure S2K). These data demonstrate that upregulation of GSDME enhances pyroptosis and radiosensitivity in NPC cells through increasing the oligomerization level of GSDME-N in vitro.

### GSDME-dependent pyroptosis sensitizes NPC to radiotherapy in vivo

**Fig. 3:**
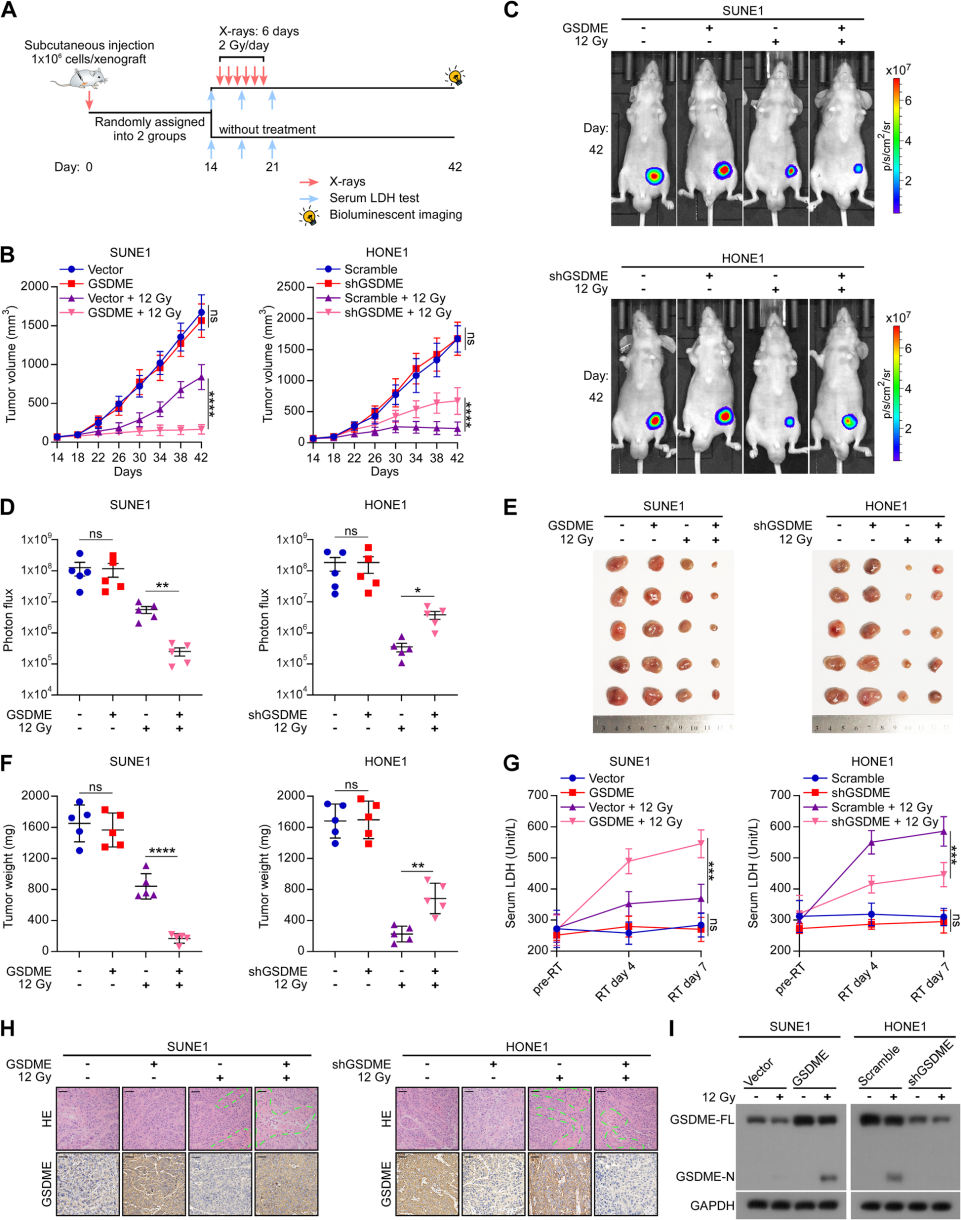
GSDME-dependent pyroptosis sensitizes NPC to radiotherapy in vivo. **A** Schematic view of the radiotherapy xenograft model. Cells stably expressing luciferase, SUNE1-Vector, SUNE1-GSDME, HONE1-Scramble, or HONE1-shGSDME, were subcutaneously injected into the nude mice (*n* = 28 mice per group). When tumor volumes reached 50–100 mm.^3^, mice bearing each type of tumor were randomized into a control group and a radiotherapy group (*n* = 14 in each subgroup). The mice in the radiotherapy group were irradiated with 12 Gy (2 Gy/day for 6 days). **B** Tumor size was monitored every 4 days (*n* = 5). **C** Representative bioluminescence images of tumors were taken on day 42. **D** Statistical analysis of photon flux (*n* = 5). **E** Images of resected tumors. **F** Tumor weight on day 42 (*n* = 5). **G** Serum LDH concentrations were determined pre-radiotherapy and following the third and sixth radiotherapy treatments (*n* = 3). **H** Representative images of HE (top) and IHC (bottom) staining of the tumor sections are shown. The dashed lines circumscribe the areas of tumor necrosis. **I** Western blotting showed the level of GSDME-N in resected tumor tissues. GAPDH was used to normalize the amount of protein loaded. Each bar represents the mean ± SD of three independent experiments. ns, No significant difference. *P < 0.05, **P < 0.01, ***P < 0.001, ****P < 0.0001

To explore whether GSDME could affect the radiosensitivity of NPC in vivo, we established xenograft mouse models. 1 × 10^6^ luciferase expressing NPC cells (SUNE1-Vector, SUNE1-GSDME, HONE1-Scramble, and HONE1-shGSDME) were subcutaneously injected into nude mice. After 14 days, the mice were randomly assigned to two groups (control group and radiotherapy group). Tumors in the pre-radiotherapy group were irradiated at a dose of 2 Gy per day (12 Gy in total). The serum LDH concentrations were detected pre-radiotherapy and after the third and sixth radiotherapy treatments. Tumor diameters were measured every four days (Fig. 3A). In the absence of radiation, no significant difference was observed in the growth rate (Fig. 3B), fluorescence signal (Fig. 3C and D), size (Fig. 3E), and weight (Fig. 3F) of tumors with upregulated or silenced GSDME compared to the controls. However, following irradiation treatment, GSDME overexpressing tumors grew distinctly slower (Fig. 3B) and exhibited weaker fluorescence signal (Fig. 3C and D), smaller size (Fig. 3E), and lower weight (Fig. 3F) compared with the vector group, while GSDME knockdown in HONE1 cells led to the opposite results. In addition, the serum LDH concentrations in the GSDME-overexpressing group were significantly higher after radiation treatment than those in the vector group, whereas the GSDME*-*knockdown group displayed lower serum LDH concentrations (Fig. 3G). H&E staining of sectioned tumors showed that overexpression of GSDME significantly increased the area of inflammatory necrosis of the tumor after irradiation, and silencing of GSDME decreased the area (Fig. 3H). Consistently, Western blotting analysis of resected tumor tissues revealed increased cleavage of GSDME in GSDME-overexpressing mice after irradiation, while silencing GSDME decreased the cleavage of GSDME (Fig. 3I). These results collectively suggest that GSDME enhances radiosensitivity of NPC cells by mediating pyroptosis in vivo.

### Low GSDME expression correlates with radioresistance and poor prognosis in NPC

**Fig. 4:**
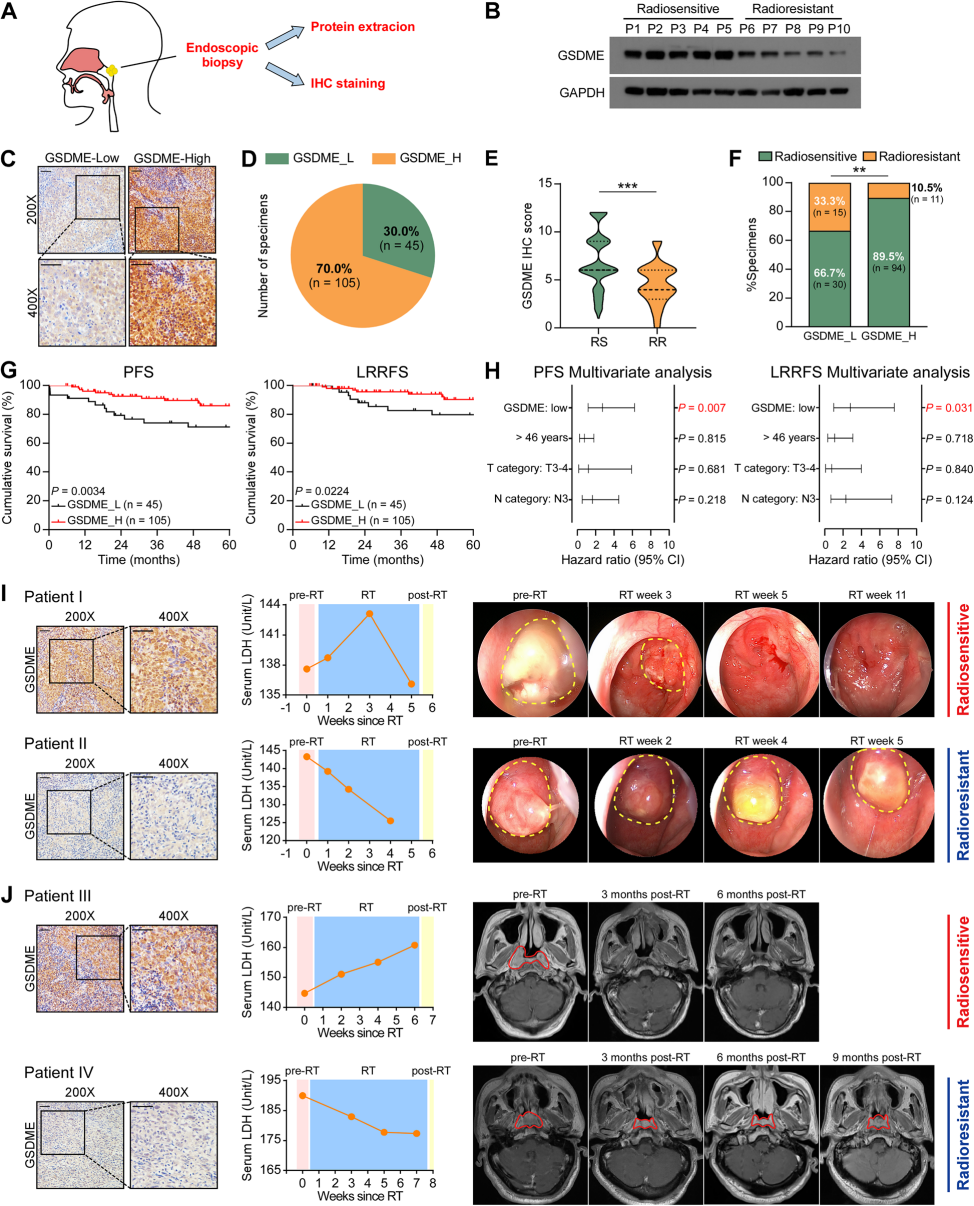
Low GSDME expression correlates with radioresistance and poor prognosis in NPC. **A** Schematic of specimens obtained from NPC via nasopharyngoscopy. **B** *GSDME* expression was analyzed by Western blotting in radiosensitive and radioresistant NPC tissues collected from 10 patients. GAPDH was used to normalize the amount of protein loaded. **C** Representative immunohistochemical images of GSDME low- and high- expression in NPC tissues (left and right, respectively). The magnified inset area is shown at the bottom. Scale bars represent 50 μm. **D** Percentage of GSDME low expression and high expression in NPC tissues (*n* = 150). Low GSDME expression, GSDME_L; High GSDME expression, GSDME_H. **E** The IHC score of GSDME in the radiosensitive and radioresistant tissues. **F** The correlation between GSDME expression and radiation therapy response. RS, radiosensitive; RR, radioresistant. **G** Association of GSDME expression with 5-year progression-free survival and 5-year locoregional recurrence-free survival was analyzed by Kaplan–Meier and Log Rank test. **H** Cox regression analysis of potential prognostic factors, including GSDME levels and other clinical characteristics. **I** Representative NPC cases received radiotherapy alone, showing the relationship between GSDME expression, serum LDH, and cancer regression. The yellow dashed lines indicate tumor in nasopharyngoscopy images. Scale bars represent 50 μm. RT, radiotherapy. **J** Representative NPC cases received chemoradiotherapy showing the relationship between GSDME expression, serum LDH, and cancer regression. The red lines indicate tumor tissue in magnetic resonance images. **P < 0.01, ***P < 0.001

To investigate whether GSDME is involved in the radiotherapy response, the level of GSDME in NPC was analyzed in 10 fresh frozen tissues and 150 paraffin-embedded tissues (Fig. 4A, Supplementary Table S1). The radioresistant NPC tissues consistently demonstrated lower protein levels of GSDME compared with radiosensitive NPC tissues (Fig. 4B). According to the scoring system described in Materials and Methods, 70.0% (105/150) of patients exhibited high GSDME expression, while 30.0% (45/150) exhibited low GSDME expression (typical low and high expression of GSDME IHC staining was presented in Fig. 4C) (Fig. 4D). The IHC score of GSDME in the radioresistant tissues was dramatically lower than that in the radiosensitive tissues (*P* = 0.0002, Fig. 4E). Subsequent analysis revealed that there was a lower percentage of radiosensitive NPC cases in the GSDME-low expression group than in the GSDME-high expression group (*P* = 0.0016, Fig. 4F). Importantly, lower GSDME expression significantly correlated with poorer PFS (*P* = 0.0034) and LRRFS (*P* = 0.0224) in all NPC cases (Fig. 4G). In addition, low GSDME expression was recognized to be a poor independent prognostic factor for 5-year PFS (*P* = 0.007) and 5-year LRRFS (*P* = 0.031) in NPC by multivariate Cox regression analysis (Fig. 4H and Supplementary Table S2). These data demonstrate that GSDME is commonly expressed in NPC, and suggest that downregulated GSDME in NPC tissues may result in radiotherapy resistance and lead to poor clinical outcomes.

Similar to previous studies on EGFR inhibitors [20], serum LDH levels were elevated after radiotherapy in a fraction of NPC subjects with high GSDME expression who showed nasopharyngoscopy-verified (Fig. 4I, patient I & Supplementary Figure S3A, patients V, VI, who received radiotherapy alone) or MR-verified (Fig. 4J, patient III & Supplementary Figure S3B, patient VIII, who received chemoradiotherapy) rapid cancer regression, representing radiosensitive cases. However, the serum LDH concentration of patients with low GSDME expression showed no apparent upward trend after radiotherapy, and nasopharyngoscopy assessment showed a slow regression of the tumor, representing radioresistance (Fig. 4I, patient II & Supplementary Figure S3A, patient VII, who received radiotherapy alone; Fig. 4J, patient IV & Supplementary Figure S3B, patients IX, X, who received chemoradiotherapy). Therefore, GSDME expression could serve as a prospective indicator of radiosensitivity in NPC patients, and the changes in serum LDH concentrations after radiotherapy could be used to monitor the efficacy of radiotherapy in these patients dynamically.

### OTUD4 deubiquitinates and stabilizes GSDME

**Fig. 5:**
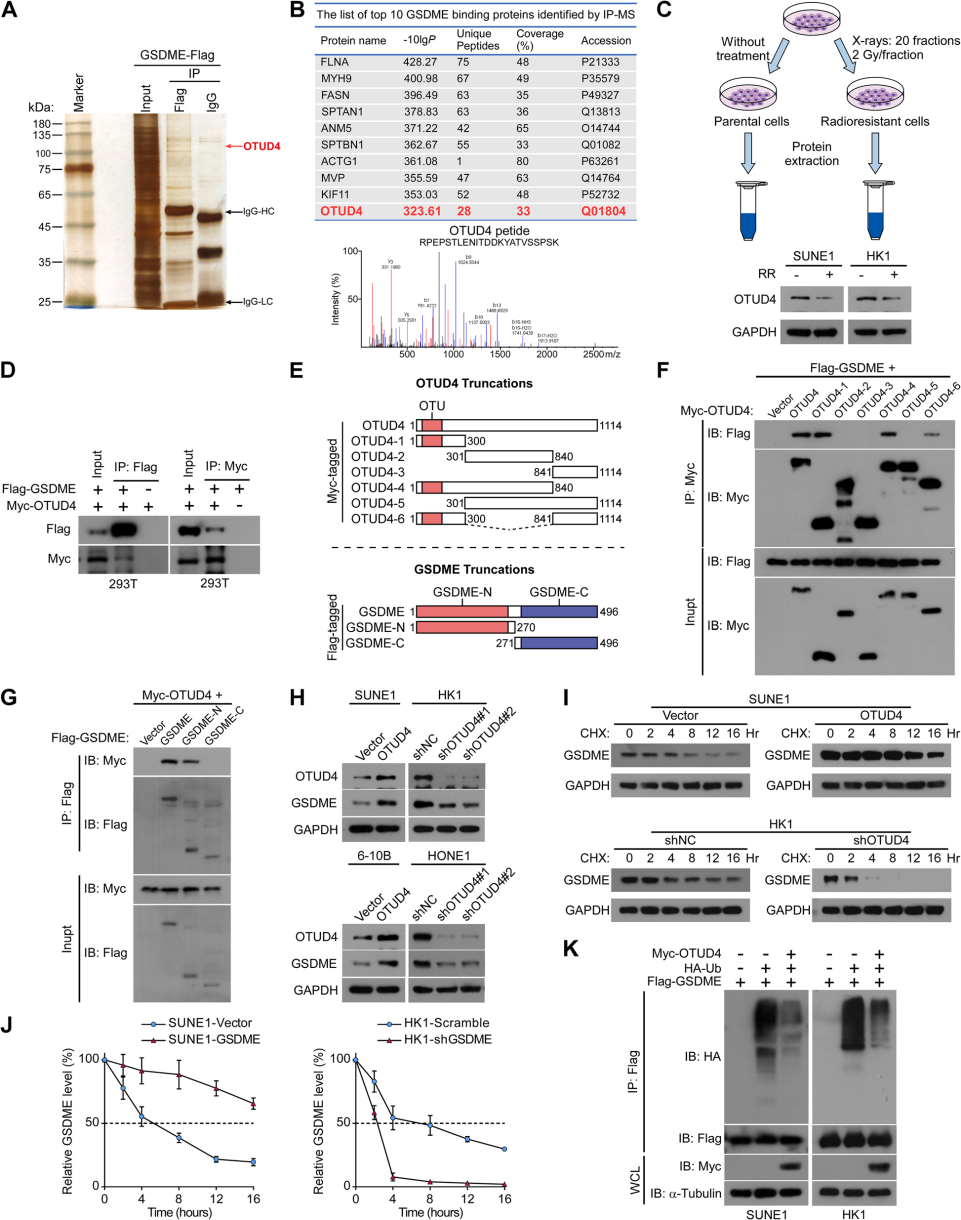
OTUD4 deubiquitinates and stabilizes GSDME. **A** Representative images of silver-stained gels of immunoprecipitation proteins using anti-IgG and anti-Flag affinity beads. **B** Upper panel: list of candidate proteins from tandem affinity purification-coupled mass spectrometry. Lower panel: MS profiles of OTUD4. **C** Upper panel: schematic of protein extraction from parental or radioresistant SUNE1 and HK1 cells. Lower panel: Western blotting showed OTUD4 protein expression in parental or radioresistant SUNE1 and HK1 cells. **D** Interaction of GSDME with OTUD4. Co-IP was performed using cell lysates obtained from HEK293T cells co-transfected with Flag-GSDME and Myc-OTUD4. **E** Upper panel: schematic of OTUD4 truncations. Lower panel: schematic of GSDME truncations. **F** HEK293T cells were transfected with Flag-GSDME and indicated Myc-OTUD4 truncations. Lysates were then IP with Myc-beads for Western blotting. **G** HEK293T cells were transfected with Myc-OTUD4 and indicated Flag-GSDME truncations. Lysates were then IP with Flag-beads for Western blotting. **H** Indicated cells were transfected with OTUD4-expressing plasmid, or infected with lentiviral mediated shRNA against OTUD4 (#1 and #2). Whole-cell extracts were collected for Western blotting analysis. **I** Cells were harvested for Western blotting analysis at indicated time points after cycloheximide (CHX) treatment. **J** Quantification of GSDME protein levels normalized to GAPDH. **K** SUNE1 and HK1 cells were transfected with HA-Ub and Flag-GSDME with or without Myc-OTUD4. After treatment with MG132 (10 µM, 6 h) GSDME ubiquitination was measured. Results are presented as mean ± SD from three independent experiments

To further explore the mechanism by which GSDME is downregulated in radioresistant NPC, real-time PCR was performed to analyze the mRNA expression of GSDME in radiosensitive and radioresistant NPC specimens. The analysis exhibited no noticeable difference in the mRNA expression of GSDME between radiosensitive and radioresistant NPC tissues (Supplementary Figure S4A). This finding indicates that GSDME downregulation may occur at the post-transcriptional level in radioresistant NPC. The ubiquitin–proteasome protein degradation pathway plays a critical role in the regulation of protein levels[37]. Interestingly, MS analysis of GSDME-interacting proteins concentrated on the deubiquitinase OTUD4 (Fig. 5A and B). Moreover, OTUD4 protein expression was distinctly down-regulated in radioresistant NPC cells (Fig. 5C). Therefore, we hypothesized that OTUD4 deubiquitylates and maintains GSDME stability. Interaction between OTUD4 and GSDME was confirmed by co-immunoprecipitation (co-IP) assays (Fig. 5D). In order to determine the regions that mediate the interaction between OTUD4 and GSDME, we made OTUD4 truncations and GSDME truncations (Fig. 5E). IP assays revealed that only truncated OTUD4 containing the OTU domain bound to GSDME, indicating that OTU domain was essential for GSDME (Fig. 5F). Moreover, the N-terminal region of GSDME was responsible for its interaction with OTUD4 (Fig. 5G). As expected, upregulation of OTUD4 distinctly increased, while OTUD4 knockdown reduced, the GSDME protein level (Fig. 5H). Upregulation of OTUD4 significantly prolonged the half-life of endogenous GSDME, and silencing OTUD4 diminished the degradation half-life of GSDME in NPC cells (Fig. 5I and J). In agreement with these findings, the overexpression of OTUD4 attenuated GSDME polyubiquitination (Fig. 5K). These results indicate that OTUD4 promotes the deubiquitination and stability of GSDME in NPC.

### OTUD4 enhances radiosensitivity in NPC cells by promoting GSDME-dependent pyroptosis

**Fig. 6:**
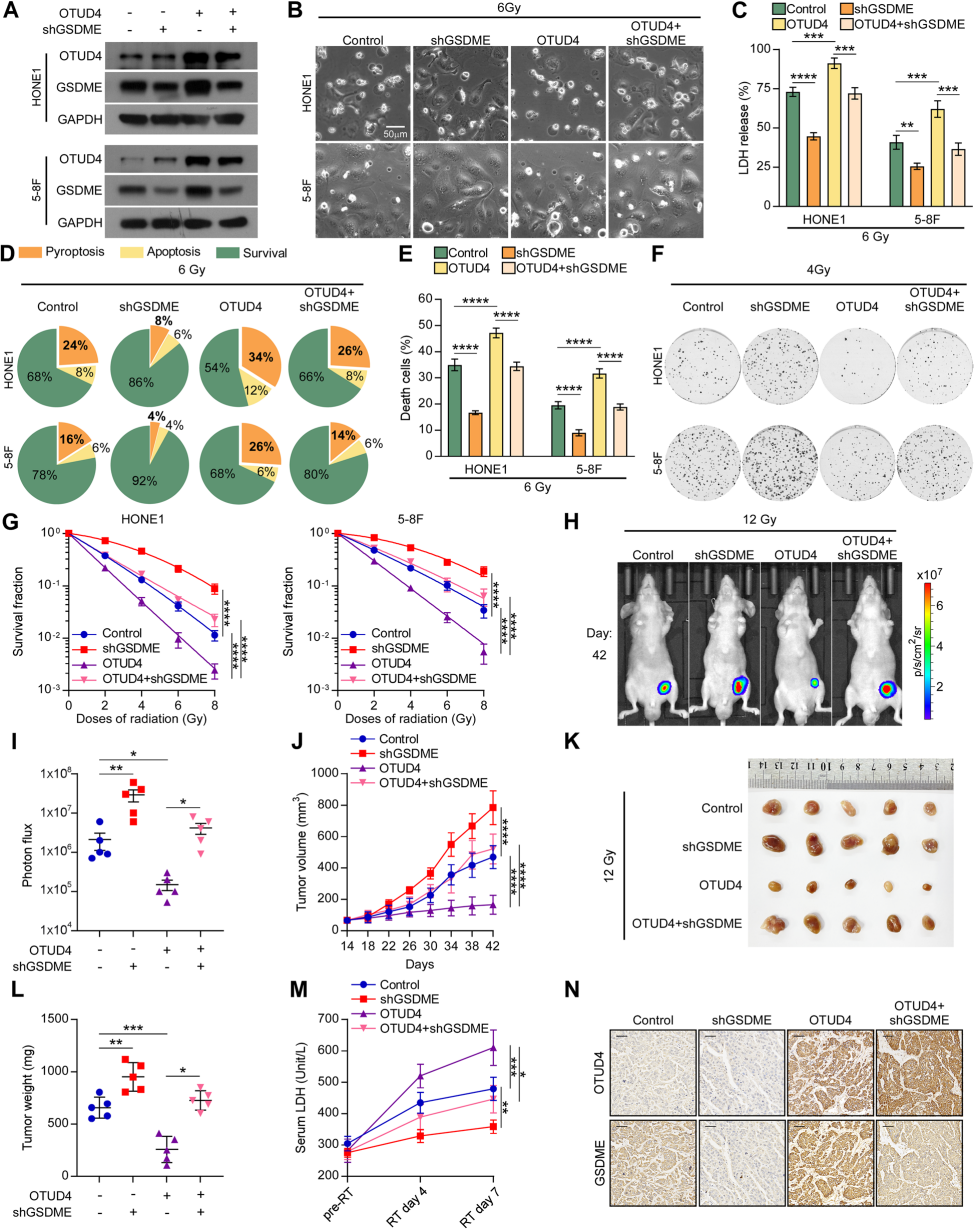
OTUD4 enhances radiosensitivity in NPC cells by promoting GSDME-dependent pyroptosis. **A** Western blotting showed the protein levels of OTUD4 and GSDME in HONE1 and 5-8F cells transfected with or without OTUD4 and shGSDME. **B-G** The above cells were exposed to the indicated irradiation dose, and then (**B**) phase-contrast cell imaging, (**C**) LDH release assay, (**D**) live cell imaging, (**E**) Annexin/PI assay, and (**F**) colony formation assay was performed at designated time points. **G** The dose-survival curves of the above cells are indicated. All data are presented as the mean ± SD of three independent experiments. **H** Representative bioluminescence images of tumors were taken on day 42. **I** Statistical analysis of photon flux (*n *= 5). **J** Tumor size was monitored every 4 days (*n* = 5). **K** Images of resected tumors. **L** Tumor weight on day 42 (*n* = 5). **M** Serum LDH concentrations were determined pre-radiotherapy and following the third and sixth radiotherapy treatments (*n* = 3). **H** Representative images of IHC staining of the tumor sections are shown. Scale bars represent 50 μm. ns, No significant difference. *P < 0.05, **P < 0.01, ***P < 0.001, ****P < 0.0001

In order to further study the impact of OTUD4 on pyroptosis and radiosensitivity in NPC cells, HONE1 and 5-8F cells with or without OTUD4 overexpression were transfected with GSDME-shRNA, and we then examined their response to ionizing radiation (Fig. 6A). OTUD4 overexpression in HONE1 and 5-8F cells led to more distinct pyroptotic bubbles (Fig. 6B), increased release of LDH (Fig. 6C), a higher proportion of pyroptosis (Fig. 6D), and a higher proportion of dead cells after irradiation (Fig. 6E; Supplementary Figure S5A). In addition, colony formation assay demonstrated that OTUD4 overexpression sensitized HONE1 and 5-8F cells to irradiation (Fig. 6F and G). However, the phenotype of OTUD4 overexpression was reversed by GSDME knockdown. In vivo experiments showed that upregulating OTUD4 or silencing GSDME did not significantly affect xenograft tumor growth without ionizing radiation (Supplementary Figure S6A-E). Nonetheless, OTUD4 overexpression sensitized xenograft tumors to irradiation, and silencing GSDME successfully impaired the effect of OTUD4 on radiosensitivity in vivo (Fig. 6H-L). In addition, serum LDH concentrations were higher in the OTUD4-overexpressing group following radiation therapy than in the control group, but silencing GSDME restored the aforementioned trend (Fig. 6M). Furthermore, IHC displayed higher GSDME protein levels in OTUD4-overexpressing tumor xenografts (Fig. 6N). More importantly, upregulating OTUD4 increased oligomerization level of GSDME-N in NPC cells after irradiation. However, the effect of OTUD4 overexpression was reversed by GSDME knockdown (Supplementary Figure S7A). These results demonstrate that OTUD4 promotes GSDME-dependent pyroptosis and enhances radiosensitivity in NPC cells by upregulating the protein level of GSDME and the oligomerization level of GSDME-N.

### The clinical relevance of the OTUD4/GSDME axis and radiotherapy response in NPC

**Fig. 7:**
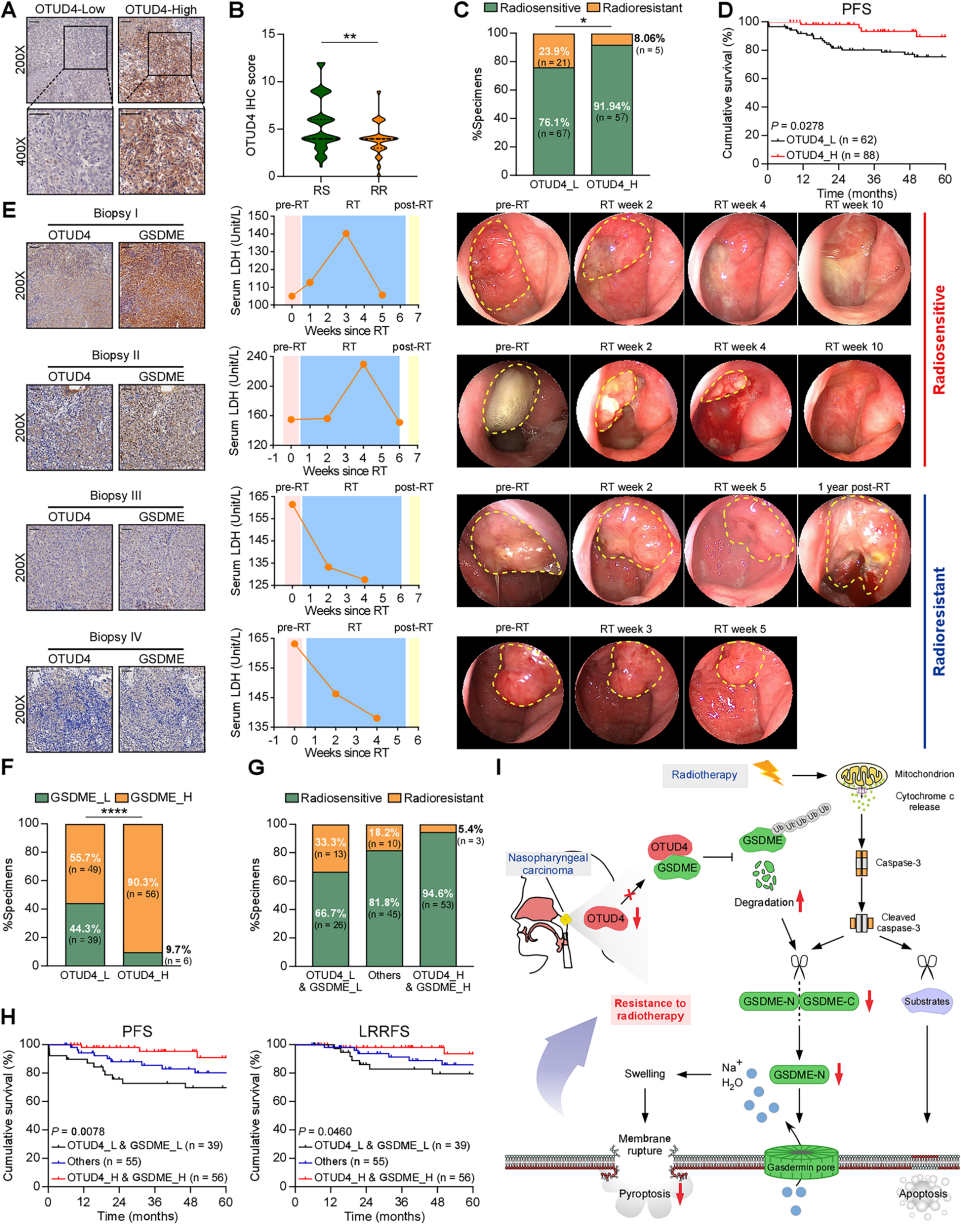
The clinical relevance of the OTUD4/GSDME axis and radiotherapy response in NPC. **A** Representative immunohistochemical images of OTUD4 low- and high- expression in NPC tissues (left and right, respectively). The magnified inset area is shown at the bottom. Scale bars represent 50 μm. **B** The IHC score of OTUD4 in the radiosensitive and radioresistant tissues. **C** The association between OTUD4 expression and radiotherapy response, χ2 test. RS, radiosensitive; RR, radioresistant. **D** 5-year PFS for NPC was calculated by Kaplan–Meier analysis, compared using the Log Rank test, and stratified by low and high OTUD4 levels. Progression-free survival, PFS. **E** Representative NPC cases received radiotherapy alone, showing the relationship between OTUD4 expression, GSDME expression, serum LDH, and cancer regression. The yellow dashed lines indicate tumor in nasopharyngoscopy images. Scale bars represent 50 μm. RT, radiotherapy. **F** The correlation between OTUD4 expression and GSDME expression. **G** The correlation between OTUD4/GSDME expression and radiosensitivity. **H** Comparison of the progression-free survival and locoregional recurrence-free survival between 39 OTUD4^low^/GSDME^low^, 56 OTUD4^high^/GSDME.^high^ and 55 others. Actuarial probabilities were analyzed by Kaplan–Meier (Log Rank test). (I) Proposed model showing that GSDME-dependent pyroptosis is identified as a critical determinant of radiosensitivity in NPC. In addition, OTUD4/GSDME interaction inhibits OTUD4-mediated GSDME ubiquitination and stability, thereby promoting GSDME-dependent pyroptosis and enhancing radiosensitivity in NPC. *P < 0.05, **P < 0.01, ****P < 0.0001

We next examined whether OTUD4 downregulation is of clinical significance and whether OTUD4 and GSDME are clinically relevant. We measured the protein level of OTUD4 in the same tissues of the 150 NPC patients. Typical low and high expression of GSDME IHC staining was presented in Fig. 7A. 41.3% (62/150) of specimens showed high OTUD4 expression, while 58.7% (88/150) exhibited low OTUD4 expression (Supplementary Figure S8A). The radioresistant group showed a lower IHC score of OTUD4 than the radiosensitive group (*P* = 0.0063, Fig. 7B). Subsequent analysis revealed that there was a lower percentage of radiosensitive NPC cases in the OTUD4-low expression group than in the OTUD4-high expression group (*P* = 0.0216, Fig. 7C). Importantly, lower OTUD4 expression significantly correlated with poorer PFS (*P* = 0.0278) in all NPC cases (Fig. 7D). These data demonstrate that downregulated OTUD4 in NPC tissues may result in radioresistance and lead to poor clinical outcomes.

More importantly, patients with low OTUD4 expression (Fig. 7E, biopsy III, IV) showed a lower level of GSDME, no significant upward trend of serum LDH concentration and a slower regression of the tumor than patients with high OTUD4 expression (Fig. 7E, biopsy I, II). Further analysis demonstrated a positive relationship between OTUD4 and GSDME expression (*P* < 0.0001, Fig. 7F, and Supplementary Table S3), validating the clinical relevance of the OTUD4/GSDME axis. More significantly, patients with both low expression of OTUD4 and low expression of GSDME had the worst radiotherapy response (66.7% vs. 81.8% vs. 94.6%, *P* = 0.0018, Fig. 7G), PFS, and LRRFS (Fig. 7H). In brief, these data further confirm that OTUD4 stabilizes and upregulates GSDME and that downregulation of OTUD4 inhibits GSDME-dependent pyroptosis, leading to radioresistance and poor prognosis in NPC.

## Discussion

Radiotherapy is the major therapeutic method for NPC; however, radioresistance is the major cause of treatment failure [38]. Previous research has focused on apoptotic deficits as a mechanism for radioresistance in NPC. In this study (summarized in Fig. 7I), we evaluated the effect of pyroptosis on the radiosensitivity of NPCs. First, we observed that pyroptosis, mediated by caspase-3 cleavage of GSDME in the mitochondrial death pathway, also plays a critical role in the radiation antitumor process in NPC. Radioresistant NPC tissues showed lower GSDME expression than radiosensitive NPC tissues. In addition, reduced GSDME expression predicted a poorer outcome and conferred NPC radioresistance both in vitro and in vivo. Furthermore, we identified GSDME as a substrate of deubiquitinase OTUD4. OTUD4 deubiquitinated and stabilized GSDME, thereby increasing GSDME-dependent pyroptosis and boosting radiosensitivity in NPC cells. More significantly, we confirmed the clinical relevance of the OTUD4/GSDME axis and radiosensitivity of NPC tissues.

Pyroptosis occurs as a result of several antitumor therapies, and contributes to the antineoplastic efficacy of these treatment modalities [9, 15–21]. For example, lobaplatin triggers pyroptosis to eliminate colon cancer cells via the ROS/JNK/Bax pathway [16]. Targeted drugs against KRAS-, EGFR- or ALK-driven lung cancer induce apoptosis and pyroptosis simultaneously [20]. Therefore, it is of great significance to investigate the potential role that pyroptosis plays in the anti-tumor activity of radiotherapy in NPC. Here we show that pyroptosis occurs in a time- and dose-dependent manner after radiotherapy. Similar to its role in the anticancer activity of other treatments [16, 17, 19, 20, 36], we suggest that pyroptosis may be a nonapoptotic death mechanism with significant clinical value in enhancing the radiosensitivity of NPC.

The gasdermin family members are indispensable pyroptosis executors [39]. In the present study we demonstrate that GSDME establishes the foundation for robust activation of pyroptosis in NPC following radiotherapy, building on the premise that radiotherapy generates considerable caspase-3 cleavage via the mitochondrial death pathway. Although GSDME is silenced in most human malignancies due to promoter methylation [33–35], GSDME is ubiquitously expressed in the majority of NPC tissues and cell lines, particularly in radiosensitive NPC. The prevalence of high GSDME expression may explain why radiotherapy is effective for the majority of NPC patients. Nevertheless, among these 150 patients, 30% of patients exhibited low expression of GSDME, indicating a poor prognosis following radiotherapy. Indeed, despite treatment with combined chemotherapy or targeted therapy with radical radiotherapy, 10% of patients with NPC have a poor prognosis and suffer from recurrence because of primary radioresistance [3, 4]. Importantly, patients with high GSDME expression exhibited elevated LDH levels and rapid tumor regression after radiotherapy, as verified by nasopharyngoscopy or MRI. Consistent with previous reports [20], we discovered that cleaved GSDME and serum LDH release by radiotherapy may represent the extent of pyroptosis; these parameters may serve as potential biomarkers for predicting radiosensitivity and improving treatment strategies.

In accordance with previous reports [20], we demonstrate that the intrinsic mitochondrial apoptotic pathway is upstream of apoptosis and GSDME-dependent pyroptosis, and can simultaneously trigger both processes. The final mode of cell death following caspase-3 cleavage is probably determined by the availability and activity of downstream effectors. However, prior research did not assess the relative contribution of pyroptosis and apoptosis to antitumor effects. In this study, live cell imaging analysis revealed that 40—75% of radiation-induced dead cells were pyroptotic cells, which contradicts the conventional view and identifies pyroptosis as a critical mode of radiotherapy-triggered tumor cell death in NPC. Notably, the ratio of pyroptotic cells to dead cells was negatively correlated with the surviving fraction, indicating that a significant pyroptosis response in NPC was associated with higher radiosensitivity. The ectopic upregulation of GSDME increased the proportion of pyroptotic cells after radiotherapy and the radiosensitivity of NPC cells.

Nanoparticles that selectively deliver decitabine directly into tumor cells reduce DNA methylation modification and elevate GSDME expression, eventually increasing chemotherapy-induced pyroptosis [40]. The identification of mechanisms that modulate GSDME expression to improve the radiotherapy response in NPC is of great significance. Since there were no significant variations in GSDME mRNA levels across radiosensitive and radioresistant patient tissues, we hypothesized that GSDME expression in NPC is regulated post-translationally. As anticipated, the mass spectrometry analysis of GSDME-interacting proteins focused on the deubiquitinase OTUD4. As a member of the OTU family, OTUD4 encodes a protein containing an OTU domain that exerts its functions through its deubiquitination activity [41]. OTUD4 maintains MAVS stability and enhances antiviral innate immune responses through cleaving K48-conjugated ubiquitin chains from MAVS [31]. The expression of OTUD4 is significantly down-regulated in various solid tumor tissues, and the upregulation of OTUD4 impedes the proliferation, migration, and invasion of liver, breast and lung cancer cells by suppressing AKT pathway [32]. In the current work, OTUD4 protein expression was revealed to be down-regulated in radioresistant NPC cells and tissues. Based on the OTU domain, OTUD4 could bind to GSDME, mediating GSDME deubiquitination and stabilization. Upregulation of OTUD4 enhances radiosensitivity in NPC cells by promoting GSDME-dependent pyroptosis. Finally, we confirmed the clinical relevance of the OTUD4/GSDME axis, and patients with low OTUD4 expression also had reduced GSDME levels. The low expression of OTUD4/GSDME is closely associated with a poor radiotherapy response.

Identifying pyroptotic cell death as the critical radiotherapy-induced cell death mechanism in NPC offers novel insights into radioresistance in NPC and suggests that the expression of GSDME influences radiosensitivity. Moreover, this study uncovered the first post-translational regulation of GSDME, which is triggered by OTUD4. Therefore, targeting OTUD4/ GSDME axis may be a promising strategy for sensitizing NPC to radiotherapy independent of apoptosis. In addition, as a mode of pro-inflammatory cell death, pyroptosis may activate the antitumor immune response [10, 21], which could also be related to enhanced radiosensitivity of NPC cells and radiotherapy-induced abscopal effects [42]. Furthermore, elucidating specific antigens and inflammatory cytokines released from radiotherapy-triggered pyroptotic tumor cells and subsequent immune responses will be helpful in guiding the combined application of immunotherapy in NPC. Finally, the discovery of GSDME as a crucial mediator of pyroptosis suggests that other potential effector proteins of pyroptosis remain to be discovered that may also be involved in modulating the response of NPC to radiotherapy.

## Conclusions

In summary, we reveal that radiotherapy elicits GSDME-dependent pyroptosis in NPC cells through the intrinsic mitochondrial apoptotic pathway, and it is a critical determinant of radiosensitivity in NPC. Additionally, OTUD4 deubiquitinates and stabilizes GSDME, resulting in increased levels of GSDME protein and radiation-induced pyroptosis, ultimately enhancing the radiosensitivity of NPC. Low expression of OTUD4 or GSDME is correlated with poor radiotherapy response and survival, while the combination of low OTUD4 and GSDME expression is associated with more significant poor prognosis. Therefore, targeting OTUD4/GSDME axis to induce pyroptosis is a novel strategy for sensitizing NPC to radiotherapy.

## Supplementary Information

**Additional file 1:** **Figure S1.** Radiotherapy induces GSDME-dependent pyroptosis in NPC cells through the intrinsic mitochondrial apoptotic pathway. **Figure S2.** Upregulation of GSDME enhances pyroptosis and radiosensitivity in NPC cells *in vitro*. **Figure S3.** Low GSDME expression correlates with radioresistance and poor prognosis in NPC. **Figure S4.** No significant difference is found in the mRNA expression of *GSDME* between radiosensitive and radioresistant NPC specimens. **Figure S5.** Knockdown GSDME significantly reverses cell dead promotion induced by overexpression OTUD4 after irradiation. **Figure S6.** Upregulating or silencing OTUD4/GSDME results in no significant difference in xenograft tumor growth in the absence of ionizing radiation. **Figure S7.** Upregulating OTUD4 increases oligomerization level of GSDME-N in NPC cells after irradiation, which is reversed by GSDME knockdown. **Figure S8.** Percentage of OTUD4 low expression and high expression in NPC tissues. **Table S1.** Clinicopathological characteristics and tumor-specific expression of GSDME in NPC. **Table S2.** Univariate and multivariate analysis of factors associated with PFS and LRRFS in 150 NPC patients. **Table S3.** Relationship between OTUD4 expression and patient clinicopathological features. **Table S4.** shRNA target sequences used in this study.

**Additional file 2: Video 1.** Representative processes of cell pyroptosis induced by 6 Gy X-ray irradiation in HNE1 cells. For time-lapse microscopy, HNE1 cells were grown on 35-mm glass bottom dishes (Nest). DIC images were acquired with a CV1000 Confocal Scanner System after irradiation.

**Additional file 3: Video 2.** Representative processes of cell apoptosis induced by 6 Gy X-ray irradiation in HNE1 cells. For time-lapse microscopy, HNE1 cells were grown on 35-mm glass bottom dishes (Nest). DIC images were acquired with a CV1000 Confocal Scanner System after irradiation.

**Additional file 4: Video 3.** Representative processes of cell survival after 6 Gy of X-ray irradiation in HNE1 cells. For time-lapse microscopy, HNE1 cells were grown on 35-mm glass bottom dishes (Nest). DIC images were acquired with a CV1000 Confocal Scanner System after irradiation.

## Data Availability

The data used and/or analyzed in the present study are available from the corresponding author on reasonable request.

## Abbreviations

NPC: Nasopharyngeal carcinoma
GSDME: Gasdermin E
OTUD4: Ovarian tumor family deubiquitinase 4
RT: Radiotherapy
IMRT: Intensity-modulated radiation therapy
DUB: Deubiquitinating enzymes
RPMI: Roswell Park Memorial Institute
FBS: Fetal bovine serum
DMEM: Dulbecco’s modified Eagle’s medium
IHC: Immunohistochemistry
LRRFS: Locoregional recurrence-free survival
PFS: Progression-free survival
ShRNAs: Short-hairpin RNAs
MTT: 3-(4,5-Dimethyl-2-thiazolyl)-2,5-diphenyl-2H-tetrazolium bromid
H&E: Hematoxylin–eosin
PARP: Poly-(ADP-ribose) polymerase
