# Supplementary material for: OTUD4-mediated GSDME deubiquitination enhances radiosensitivity in nasopharyngeal carcinoma by inducing pyroptosis
Source: J Exp Clin Cancer Res. 2022 Nov 21;41:328. doi: 10.1186/s13046-022-02533-9 (PMC9677691; doi:10.1186/s13046-022-02533-9)
Supplement: Supplementary file 1 — Additional file 1: Figure S1. Radiotherapy induces GSDME-dependent pyroptosis in NPC cells through the intrinsic mitochondrial apoptotic pathway. Figure S2. Upregulation of GSDME enhances pyroptosis and radiosensitivity in NPC cells in vitro. Figure S3. Low GSDME expression correlates with radioresistance and poor prognosis in NPC. Figure S4. No significant difference is found in the mRNA expression of GSDME between radiosensitive and radioresistant NPC specimens. Figure S5. Knockdown GSDME significantly reverses cell dead promotion induced by overexpression OTUD4 after irradiation. Figure S6. Upregulating or silencing OTUD4/GSDME results in no significant difference in xenograft tumor growth in the absence of ionizing radiation. Figure S7. Upregulating OTUD4 increases oligomerization level of GSDME-N in NPC cells after irradiation, which is reversed by GSDME knockdown. Figure S8. Percentage of OTUD4 low expression and high expression in NPC tissues. Table S1. Clinicopathological characteristics and tumor-specific expression of GSDME in NPC. Table S2. Univariate and multivariate analysis of factors associated with PFS and LRRFS in 150 NPC patients. Table S3. Relationship between OTUD4 expression and patient clinicopathological features. Table S4. shRNA target sequences used in this study. [file 13046_2022_2533_MOESM1_ESM.docx]

**Supplementary Information**

**Supplementary Materials and Methods**

***Extraction of RNA, reverse transcription, and quantitative real-time PCR***

Trizol (Invitrogen, Carlsbad, CA, USA) was used to isolate total RNA from the clinical tissues. Random hexamer primers were then used to prime cDNA synthesis from 1 μg of RNA. Quantitative real-time PCR was performed using FastStart Universal SYBR Green Master (ROX; Roche, Toronto, ON, Canada) on a CFX96 Real Time System C1000 Cycler (Bio-Rad Laboratories, Singapore). All reactions were incubated at 95 °C for 3 min, followed by 40 cycles of 95 °C for 15 s, 60 °C for 15 s, and 72°C for 7 min. Primer sequences were as follows: *GSDME* forward 5′- GATCTCTGAGCACATGCAGGTC -3′ and reverse 5′- GTTGGAGTCCTTGGTGACATTCC -3′; Glyceraldehyde-3-phosphate dehydrogenase (GAPDH) forward 5′-AAGGTCATCCCTGAGCTGAA-3′ and reverse 5′-TGACAAAGTGGTCGT TGAGG-3′. The expression of GSDME in the samples was normalized to that of *GAPDH*; these experiments were performed at least in triplicate.

***Radiation treatment***

For *in vitro* irradiation, cells were displayed to X-rays using an X-ray irradiator RS2000 (1.1 Gy/min, 160 kV; RAD SOURCE, USA). For *in vivo* irradiation, mice were firstly anesthetized, then placed in a box, and finally fixed in place. The subcutaneous tumor was settled within the center of the irradiation field. Only the tumor was exposed to irradiation and lead was used to shield the other parts of the mouse.

***Colony formation assay***

For attachment, cells were seeded in 6-well plates and cultured at 37 °C for 24 h, before being irradiated with 0, 2, 4, 6, or 8 Gy using X-rays. After approximately 10-14 days, cell colonies were washed with phosphate-buffered saline (PBS), and fixed with 4% formaldehyde. After staining with 0.05% crystal violet, the number of colonies that comprised at least 50 cells was counted using an inverted microscope. The surviving fraction (SF) was calculated as: Number of colonies / (number of cells seeded × plating efficiency), where the plating efficiency (PE) was the number of control colonies obtained divided by the number of control cells seeded. A dose survival curve was calculated and fitted with a linear-quadratic model using GraphPad Prism 5.0 Software (GraphPad Software Inc., La Jolla, CA, USA).

***Polyubiquitination analysis***

Myc-OTUD4, HA-Ub, and Flag-GSDME were transfected into SUNE1 and 6-10B cells with Lipofectamine 3000 reagent for 24 h. Cells were then treated with MG132 (10 µM) for 6 h, harvested, and lysed in IP lysis buffer (150 mM NaCl, 10 mM HEPES, pH 7.4, 1% NP-40). The cell lysates were precipitated with anti-Flag-tag beads (Sigma-Aldrich) overnight at 4°C. Precipitates were washed three times in HEPES buffer and were solubilized with 2X sample buffer. The proteins were analyzed by western blot analysis using anti-HA tag antibody.

**Supplementary figures and figure legends**

**
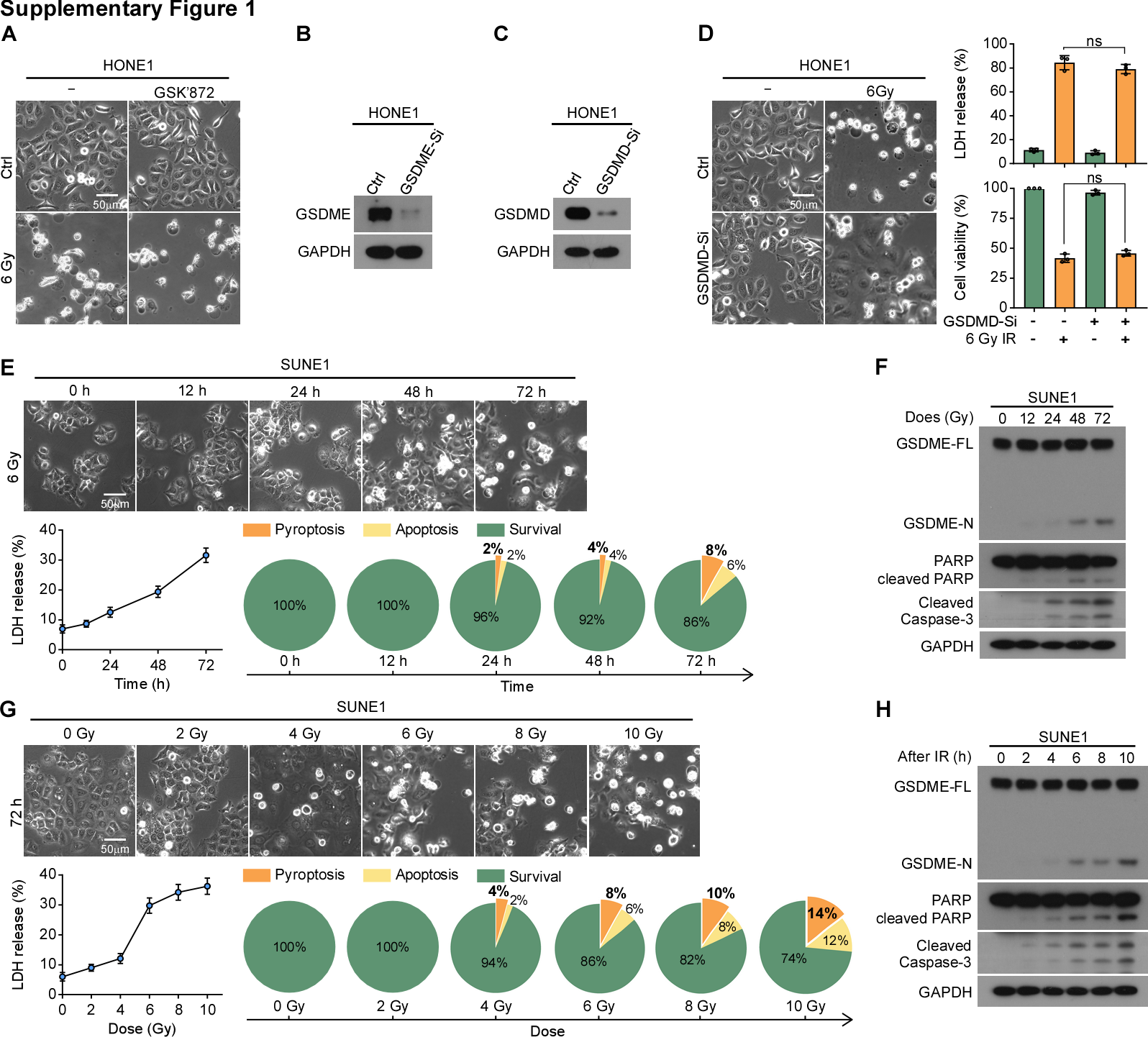
Figure S1. Radiotherapy induces GSDME-dependent pyroptosis in NPC cells through the intrinsic mitochondrial apoptotic pathway.** (A) Necroptosis inhibitor GSK’872 had no effect on radiation-induced pyroptosis in HONE1 cells at 72 hours after 6 Gy X-rays irradiation. (B) GSDME knockdown efficiency in HONE1 cells. (C) GSDMD knockdown efficiency in HONE1 cells. (D) Knockdown of GSDMD had no effect on radiation-induced pyroptosis (left), LDH release (upper right), and cell death (bottom right) in HONE1 cells at 72 hours after 6 Gy X-rays irradiation. (E) Cell morphological changes (top), LDH release assay (bottom left), live cell imaging (bottom right), and (F) the levels of indicated proteins were assessed in SUNE1 cell lines at the indicated time points after irradiation (6 Gy). (G) Cell morphological changes (top), LDH release assay (bottom left), live cell imaging (bottom right), and (H) the levels of indicated proteins were assessed in SUNE1 cell lines at 72 hours after the indicated irradiation dose. GAPDH was used to normalize the amount of protein loaded. All data are presented as the mean ± SD of three independent experiments. ns, No significant difference.

**
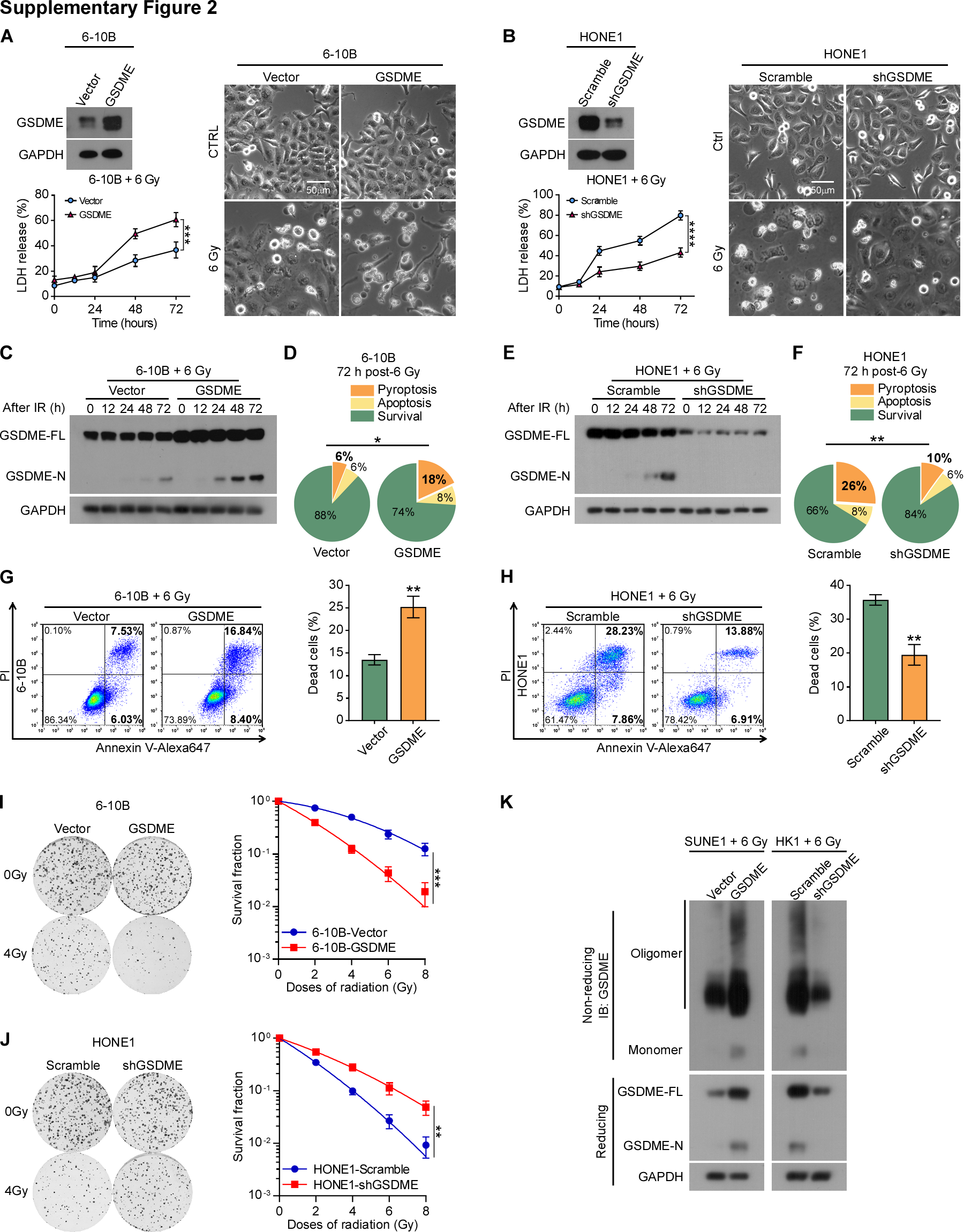
Figure S2. Upregulation of GSDME enhances pyroptosis and radiosensitivity in NPC cells *in vitro*.** (A-J) Stable GSDME-overexpressing 6-10B cells (A, left upper) and stable GSDME-knockdown HONE1 cells (B, left upper) were established. These cells were exposed to the indicated irradiation dose, and then phase-contrast cell imaging (A, B, right), LDH release assay (A, B, left lower), western blot analysis of GSDME-N (C, E), live cell imaging (D, F), Annexin/PI assay (G, H), and colony formation assay (I, J, left) were performed at designated time points. The survival curves of stable cell lines are indicated (I, J, right). (K) NPC cells expressing the vector, GSDME, scramble or shGSDME were exposed to irradiation at 6 Gy dose. Cells were lysed in RIPA buffer and then mixed with loading buffer with or without β-mercaptoethanol and subjected to immunoblotting analysis. GAPDH was used to determine the amount of loading proteins. All data are presented as the mean ± SD of three independent experiments. **P*< 0.05, ***P*< 0.01, ****P*< 0.001, *****P*< 0.0001.

**
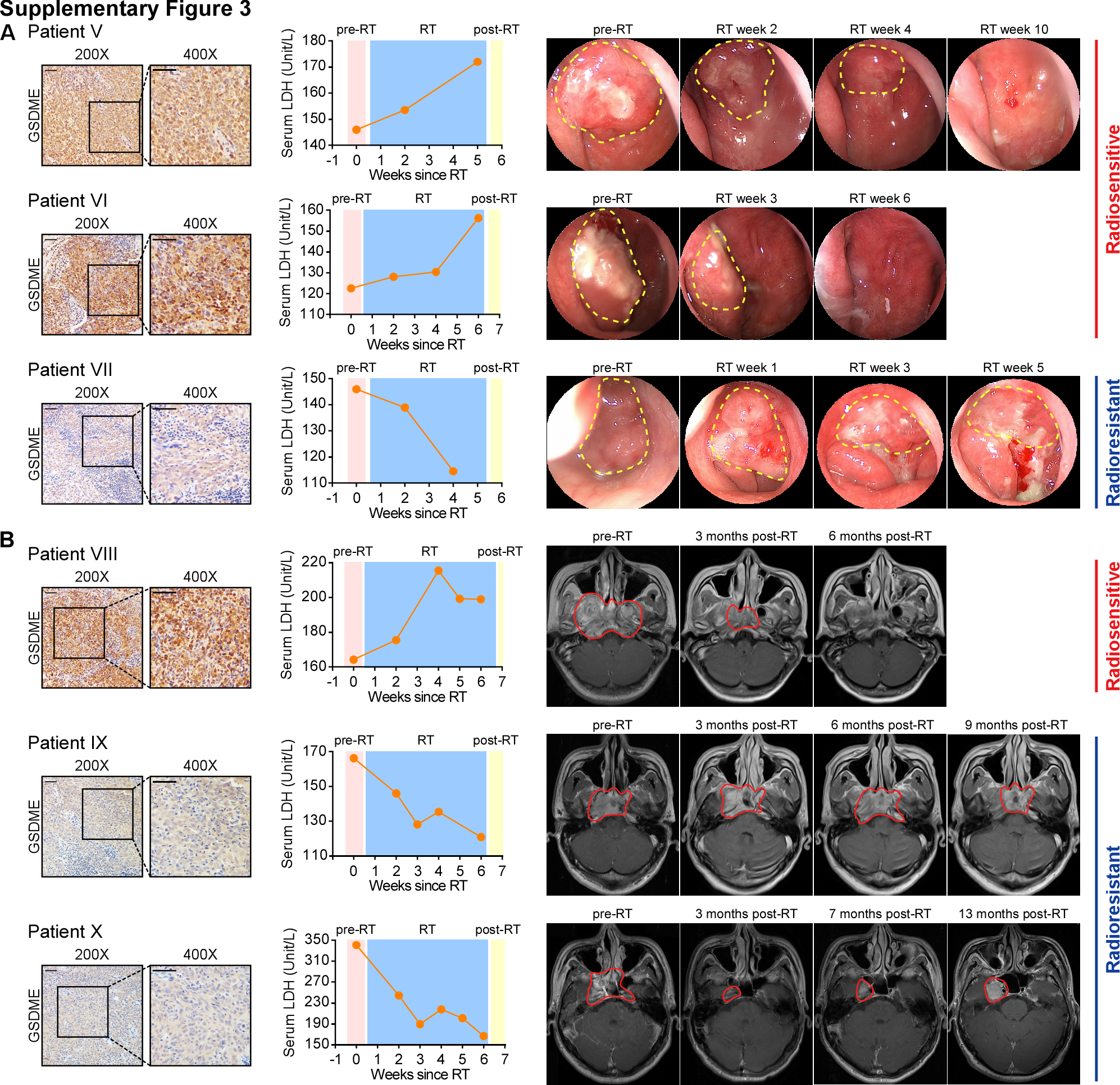
Figure S3. Low GSDME expression correlates with radioresistance and poor prognosis in NPC.** (A) Representative NPC cases received radiotherapy alone, showing the relationship between GSDME expression, serum LDH, and cancer regression. The yellow dashed lines indicate tumor in nasopharyngoscopy images. Scale bars represent 50 μm. RT, radiotherapy. (B) Representative NPC cases received chemoradiotherapy showing the relationship between GSDME expression, serum LDH, and cancer regression. The red lines indicate tumor tissue in magnetic resonance images.

**
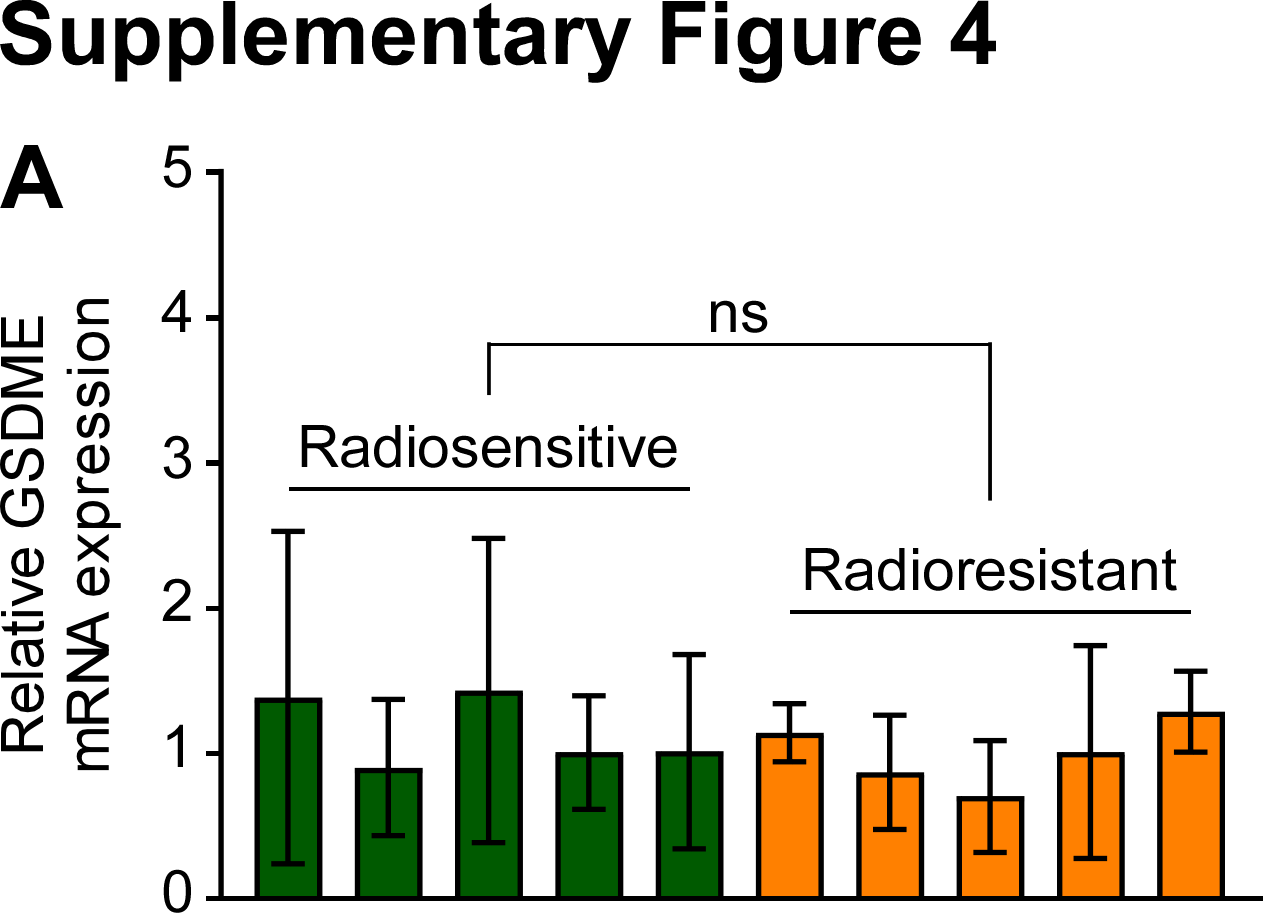
**

**Figure S4.** **No significant difference is found in the mRNA expression of *GSDME* between radiosensitive and radioresistant NPC specimens.** (A) RT-PCR analysis of GSDME mRNA expression in pretreatment biopsy specimens from 10 NPC patients. Data represent the mean ± SD of triplicates, Mann–Whitney test. ns, No significant difference.

**
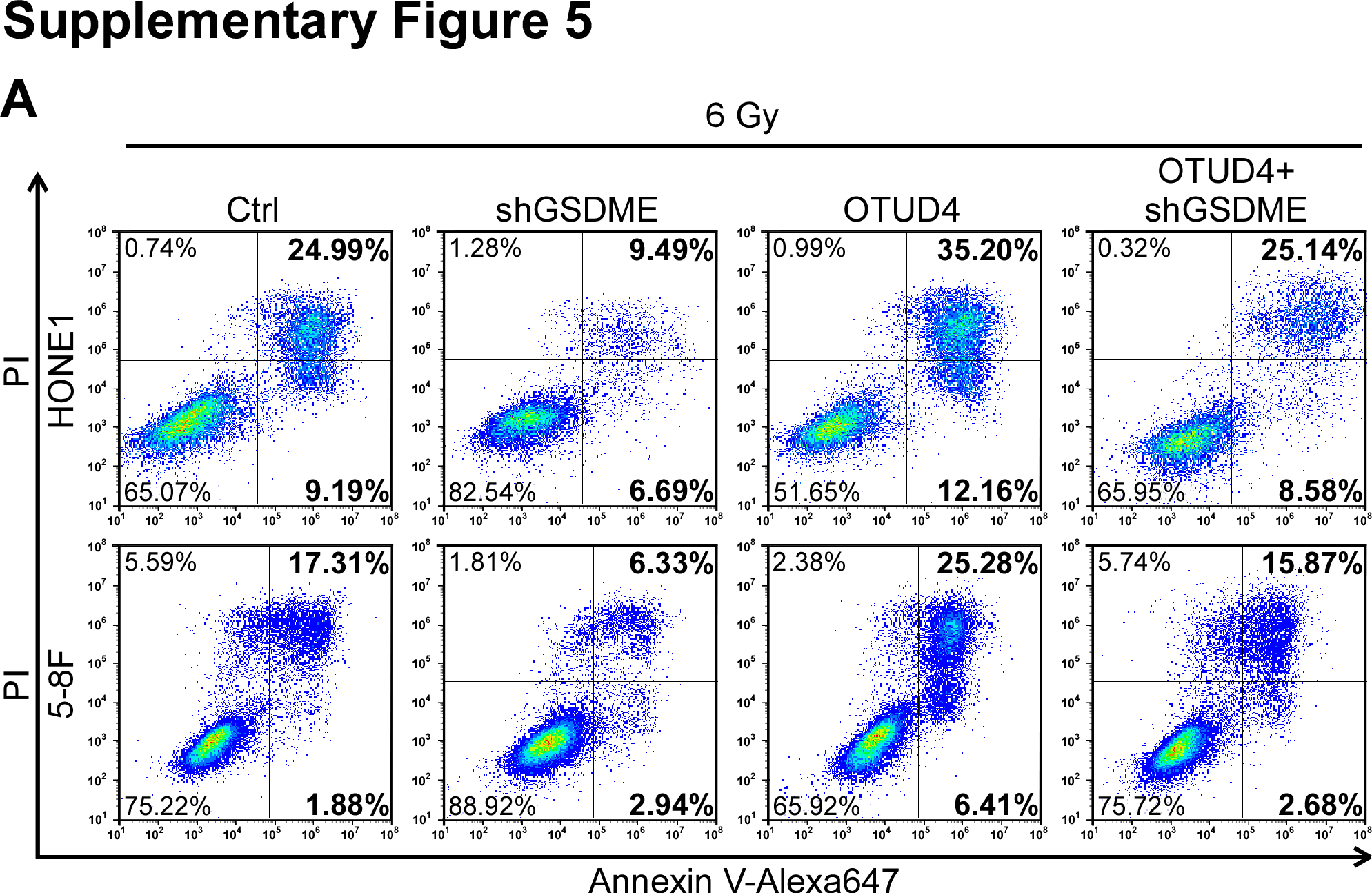
**

**Figure S5. Knockdown GSDME significantly reverses cell dead promotion induced by overexpression OTUD4 after irradiation.** (A) Representative results of Annexin/PI assay by flow cytometry in HONE1 and 5-8F cells transfected with or without OTUD4 and shGSDME.

**
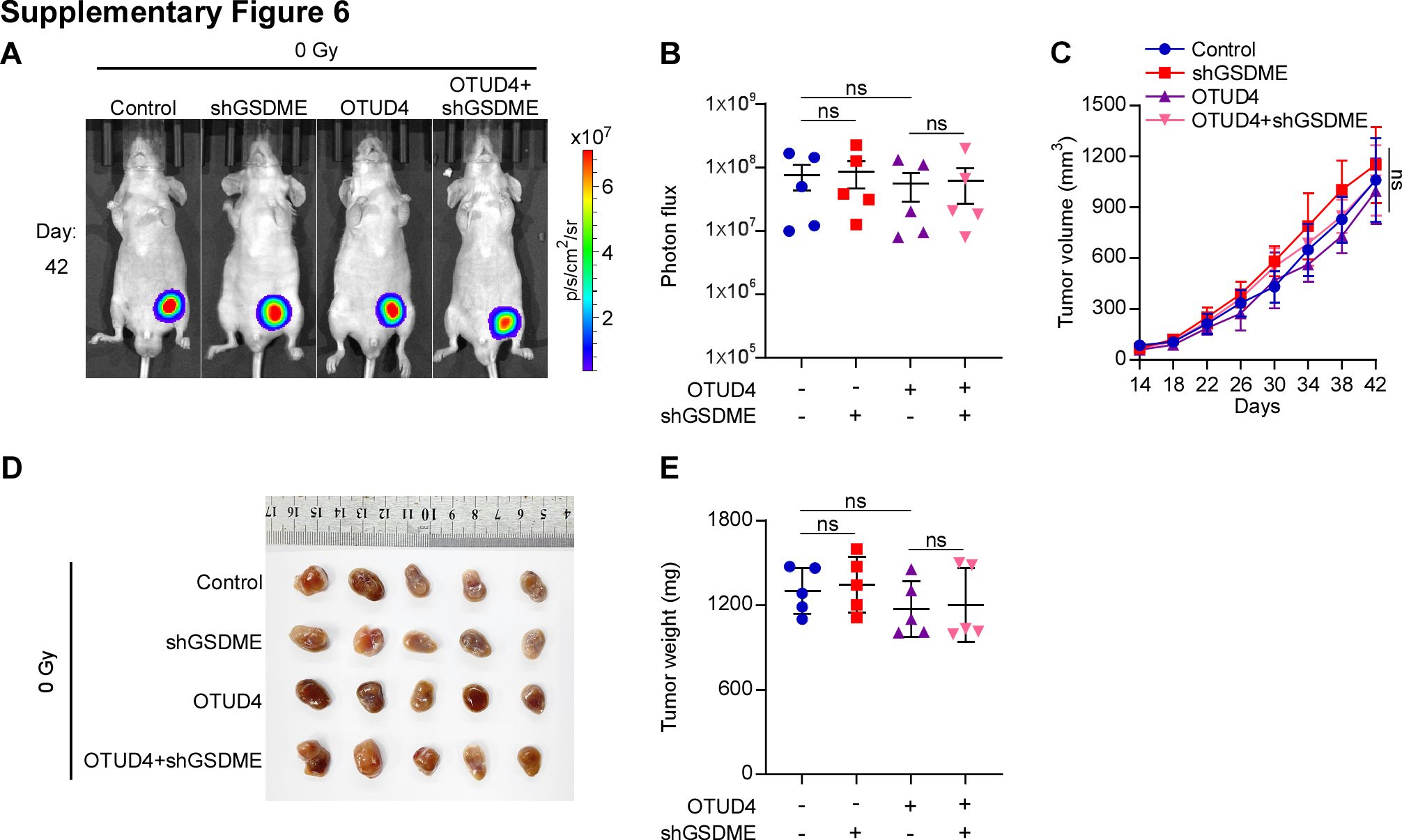
 Figure S6. Upregulating or silencing OTUD4/GSDME results in no significant difference in xenograft tumor growth in the absence of ionizing radiation.** (A) Representative bioluminescence images of tumors in different groups in the absence of ionizing radiation. (B) Statistical analysis of photon flux (n = 5). (C) Tumor diameters were measured every 4 days (n = 5). (D) Images of resected tumors. (E) Tumor weight on day 42 (n = 5). ns, No significant difference.

**
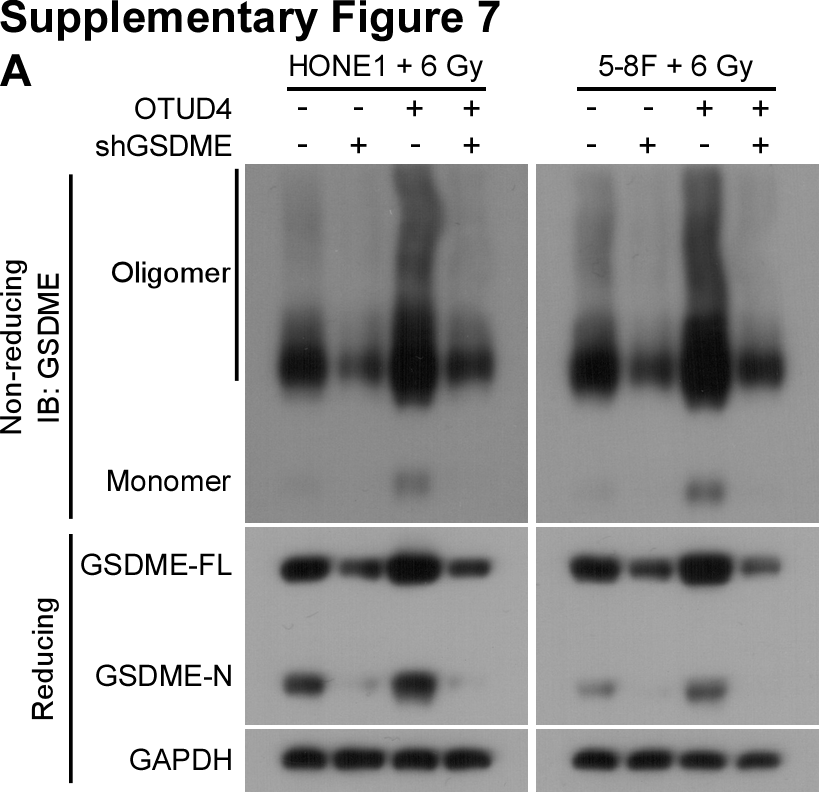
**

**Figure S7. Upregulating OTUD4 increases oligomerization level of GSDME-N in NPC cells after irradiation, which is reversed by GSDME knockdown.** (A) HONE1 and 5-8F cells transfected with or without OTUD4 and shGSDME were exposed to irradiation at 6 Gy dose. Cells were lysed in RIPA buffer and then mixed with loading buffer with or without β-mercaptoethanol and subjected to immunoblotting analysis. GAPDH was used to determine the amount of loading proteins.


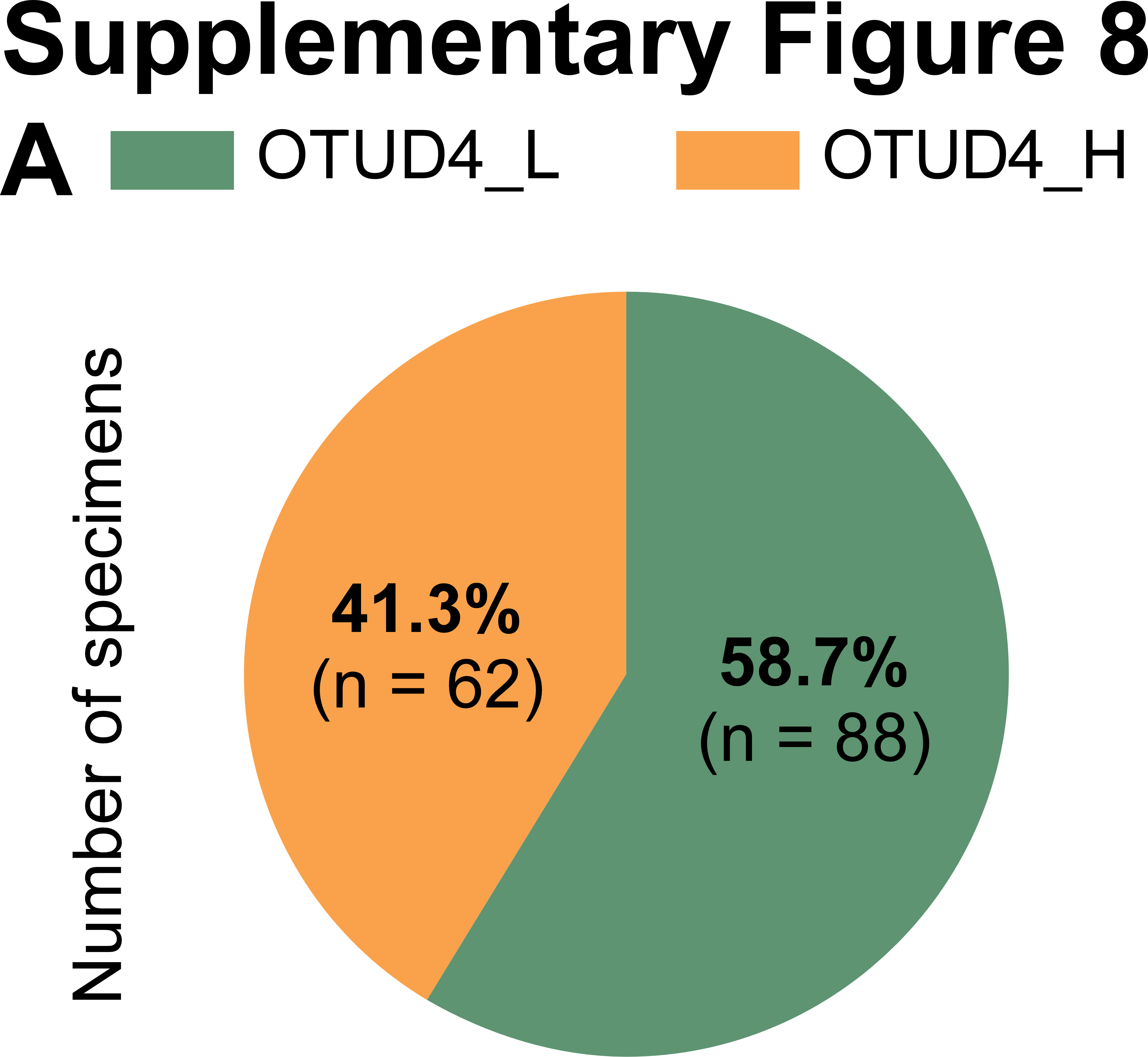


**Figure S8. Percentage of OTUD4 low expression and high expression in NPC tissues.** (A) Percentage of OTUD4 low expression and high expression in NPC tissues (n = 150). Low OTUD4 expression, OTUD4_L; High OTUD4 expression, OTUD4_H.

**Supplementary tables**

**Table S1. Clinicopathological characteristics and tumor-specific expression of GSDME in NPC.**

| **Parameters** | **N (%)** |
| --- | --- |
| **Sex** |  |
| Male | 121 (80.7) |
| Female | 29 (19.3) |
| **Age, years** |  |
| ≤46 | 75 (50.0) |
| >46 | 75 (50.0) |
| **WHO category** |  |
| II | 2 (1.3) |
| III | 148 (98.7) |
| **T classification** |  |
| T1-2 | 23 (15.3) |
| T3-4 | 127 (84.7) |
| **N classification** |  |
| N0-2 | 128 (85.3) |
| N3 | 22 (14.7) |
| **Clinical stage** |  |
| I-II | 11 (7.3) |
| III-IV | 139 (92.7) |
| **Locoregional recurrence** |  |
| No | 133 (88.7) |
| Yes | 17 (11.3) |
| **Distant metastasis** |  |
| No | 134 (89.3) |
| Yes | 16 (10.7) |
| **Vital status** |  |
| Alive | 141 (94.0) |
| Dead | 9 (6.0) |
| **GSDME expression** |  |
| Low | 45 (30.0) |
| High | 105 (70.0) |

Abbreviations: WHO, World Health Organization; II: differentiated non-keratinized carcinoma; III: undifferentiated non-keratinized carcinoma; T, tumor; N, node; GSDME, gasdermin E.

**Table S2.** **Univariate and multivariate analysis of factors associated with PFS and LRRFS in 150 NPC patients**

| **Characteristics** | **Univariate analysis** | | **Multivariate analysis** | |
| --- | --- | --- | --- | --- |
|  | **HR (95% CI)** | ***P*** | **HR (95% CI)** | ***P*** |
| **PFS** |  |  |  |  |
| GSDME (low vs. high) | 3.026 (1.388-6.598) | **0.005** | 2.908 (1.330-6.358) | **0.007** |
| Age (> 46 vs. ≤46) | 0.881 (0.407-1.906) | 0.748 | 0.911 (0.419-1.982) | 0.815 |
| T category (T3-4 vs. T1-2) | 1.269 (0.297-5.414) | 0.747 | 1.365 (0.309-6.041) | 0.681 |
| N category (N3 vs. N0-2) | 1.863 (0.746-4.655) | 0.183 | 1.813 (0.703-4.672) | 0.218 |
| **LRRFS** |  |  |  |  |
| GSDME (low vs. high) | 2.930 (1.114-7.707) | **0.029** | 2.911 (1.102-7.690) | **0.031** |
| Age (> 46 vs. ≤46) | 1.193 (0.460-3.094) | 0.716 | 1.195 (0.454-3.147) | 0.718 |
| T category (T3-4 vs. T1-2) | 0.637 (0.145-2.808) | 0.552 | 0.851 (0.178-4.074) | 0.840 |
| N category (N3 vs. N0-2) | 2.664 (0.934-7.598) | 0.067 | 2.410 (0.786-7.387) | 0.124 |

HR, hazard ratio; CI, confidence interval; PFS, progression-free survival; LRRFS, locoregional recurrence-free survival.

**Table S3. Relationship between OTUD4 expression and patient clinicopathological features.**

| **Factors** | **OTUD4 expression** | | ***P*-value** |
| --- | --- | --- | --- |
|  | **Low (%)** | **High (%)** |  |
| **Sex** |  |  |  |
| Male | 70 (57.9) | 51 (42.1) |  |
| Female | 18 (62.1) | 11 (37.9) | 0.679 |
| **Age, years** |  |  |  |
| ≤46 | 46 (61.3) | 29 (38.7) |  |
| >46 | 42 (56.0) | 33 (44.0) | 0.507 |
| **WHO category** |  |  |  |
| II | 1 (50.0) | 1 (50.0) |  |
| III | 87 (58.8) | 61 (40.7) | 0.802 |
| **T classification** |  |  |  |
| T1-2 | 13 (56.5) | 10 (43.5) |  |
| T3-4 | 75 (59.1) | 52 (40.9) | 0.820 |
| **N classification** |  |  |  |
| N0-2 | 76 (59.4) | 52 (40.6) |  |
| N3 | 12 (54.5) | 10 (45.5) | 0.671 |
| **Clinical stage** |  |  |  |
| I-II | 7 (63.6) | 4 (36.4) |  |
| III-IV | 81 (58.3) | 58 (41.7) | 0.728 |
| **Locoregional recurrence** |  |  |  |
| No | 74 (55.6) | 59 (44.4) |  |
| Yes | 14 (82.4) | 3 (17.6) | 0.035 |
| **Distant metastasis** |  |  |  |
| No | 75 (56.0) | 59 (44.0) |  |
| Yes | 13 (81.2) | 3 (18.8) | 0.052 |
| **Vital status** |  |  |  |
| Alive | 80 (56.7) | 61 (43.3) |  |
| Dead | 8 (88.9) | 1 (11.1) | 0.058 |
| **GSDME expression** |  |  |  |
| Low | 39 (86.7) | 6 (13.3) |  |
| High | 49 (46.7) | 56 (53.3) | 0.000 |

**Table S4. shRNA target sequences used in this study.**

| shRNA sequence | |
| --- | --- |
| shGSDME#1 | GATGATGGAGTATCTGATCTT |
| shOTUD4#1 | GCGTTTATAGAAGGATCATTT |
| shOTUD4#2 | CACTATAGATTCCAAACATAA |
